# Three‐in‐One Zinc Anodes Created by a Large‐scale Two‐Step Method Achieving Excellent Long‐Term Cyclic Reversibility and Thin Electrode Integrity

**DOI:** 10.1002/advs.202401575

**Published:** 2024-05-20

**Authors:** Hongfei Lu, Di Zhang, Zhenjie Zhu, Nawei Lyu, Xin Jiang, Chenxu Duan, Yi Qin, Xinyao Yuan, Yang Jin

**Affiliations:** ^1^ Research Center of Grid Energy Storage and Battery Application School of Electrical and Information Engineering Zhengzhou University Zhengzhou Henan 450001 China

**Keywords:** coating, electroplating, zinc anode, zinc deposition, zinc‐ion batteries

## Abstract

Practical aqueous zinc‐ion batteries require low‐cost thin zinc anodes with long‐term reversible stripping/depositing. However, thin zinc anodes encounter more severe issues than thick zinc, such as dendrites and uneven stripping, resulting in subpar performance and limited lifetimes. Here, this work proposes a three‐in‐one zinc anode obtained by a large‐scale two‐step method to address the above issues. In a three‐in‐one zinc anode, the copper foil as an inactive current collector solves the gradual reduction of the active area when only the pure zinc as an active current collector. This work develops an automatic electroplating device that can continuously deposit a zinc layer on a conducting foil to meet the demand for zinc‐coated copper foils. The sodium carboxymethylcellulose (CMC)‐zinc fluoride (ZnF_2_) protective layer prevents direct contact between zinc and separator, and provides a uniform and sufficient supply of zinc ions. The CMC‐ZnF_2_‐coated copper foil performs up to 3000 reversible zinc deposition/stripping cycles with a cumulative capacity of 6 Ah cm^−2^ and an average Coulombic efficiency of 99.94%. The Zn||ZnVO cell using the three‐in‐one anode achieved a high capacity retention of over 70% after 15 000 cycles. The proposed three‐in‐one anode and the automatic electroplating device will facilitate industrialization of practical thin zinc anodes.

## Graphical abstract

This work reports a Cu‐based Zn anode with a CMC‐ZnF_2_ coating. This three‐in‐one electrode can be mass‐produced by a two‐step “electroplating and coating” process, where step I is accomplished using an automatic electroplating device. Thanks to the inactive current collector and the protective layer, the anode maintains integrity and deposition uniformity during battery cycling, thereby improving performance and life.

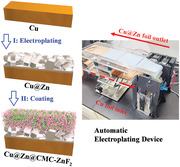

## 1 Introduction

In recent years, aqueous zinc‐ion batteries have been favored for flexible wearable devices and large‐scale stationary energy storage due to their intrinsic safety, low cost and other advantages.^[^ ^1^, ^2^ ^]^ The global renewable energy capacity witnessed a remarkable surge in 2023, reaching an impressive 510 gigawatts, with China accounting for over 50% of this expansion, according to the official website of the National Energy Administration of China. The booming development of energy storage has also increased the pursuit of the batteries with intrinsic safety performance,^[^ ^3^ ^]^ such as zinc‐ion batteries. By leveraging their advantages in terms of high safety and low cost, aqueous zinc‐ion batteries may eventually catch up with lithium‐ion batteries in terms of market share for energy storage. However, one of the critical challenges lies in the reduced significantly life caused under the high depth‐of‐discharge (DOD) of zinc anodes which hampers practical application.^[^ ^1^, ^4^ ^]^

In order to achieve practical zinc‐ion batteries, it is necessary to utilize thinner zinc foils.^[^ ^5^ ^]^ For example, when using a 200 µm Zn foil with an approximately weight of 107.15 mg cm^−2^, its theoretical capacity is ≈87.86 mAh cm^−2^ (Table S1, Supporting Information). When it paired with a cathode possessing a capacity of 5 mAh cm^−2^, the DOD for the 200 µm Zn foil amounts to ≈5.7%. Conversely, for thinner Zn foils such as 30, 20,^[^ ^6^ ^]^ and 10 µm Zn foils, their theoretical capacities are ≈16.10, 13.06, and 8.05 mAh cm^−2^, respectively (Table S1, Supporting Information). Additionally, when paired with a cathode possessing a capacity of 5 mAh cm^−2^, their corresponding DODs are ≈31.1%, 38.3%, and 62.1%. Nevertheless, the problems of zinc dendrites and uneven stripping are more severe for thinner zinc anodes, resulting in possible short‐circuiting and partial complete stripping. Therefore, it is essential to enhance the cyclic reversibility of stripping/plating or plating/stripping for thin zinc anodes.^[^ ^7^ ^]^

Currently, numerous effective strategies have existed to enhance the reversibility of zinc metal stripping/depositing or depositing/stripping, such as bulk zinc optimization,^[^ ^8^, ^9^, ^10^, ^11^ ^]^ coatings,^[^ ^12^, ^13^, ^14^, ^15^, ^16^, ^17^ ^]^ separators,^[^ ^18^, ^19^ ^]^ electrolyte additives,^[^ ^20^, ^21^, ^22^, ^23^, ^24^, ^25^ ^]^ high‐concentration electrolytes,^[^ ^26^, ^27^ ^]^ nanomicellar electrolyte,^[^ ^28^ ^]^ and gradient designs.^[^ ^29^, ^30^ ^]^ Moreover, copper foils utilized in anode‐free zinc‐ion batteries^[^ ^31^, ^32^, ^33^ ^]^ for achieving a desirable 100% DOD possess several advantages including cost‐effectiveness, excellent ductility and conductivity, suitable electrode potential, and low zinc deposition barrier. However, anode‐free zinc‐ion batteries encounter two major challenges. First, it is challenging to find zinc‐abundant cathodes with excellent electrical conductivity and high specific capacity. Cathodes like V_2_O_5_, MnO_2_,^[^ ^34^ ^]^ and Zn_0.25_V_2_O_5_·nH_2_O (ZnVO)^[^ ^35^ ^]^ that we are familiar with cannot be directly employed in anode‐free zinc‐ion batteries. Second, the discharge specific capacity of the battery at 100% DOD falls short of our expectations. For example, in a modified anode‐free zinc‐ion battery system utilizing Zn_3_V_3_O_8_ as a cathode only exhibits a specific capacity of ≈70 mAh g^−1^ while employing pure Zn as an anode enables the Zn_3_V_3_O_8_ cathode to achieve ≈300 mA h g^−1^ at a higher current density of 2 A g^−1^.^[^ ^33^ ^]^ Thus the anode‐free zinc‐ion batteries at current levels fail to improve the energy density of zinc‐ion batteries.

**Figure 1:**
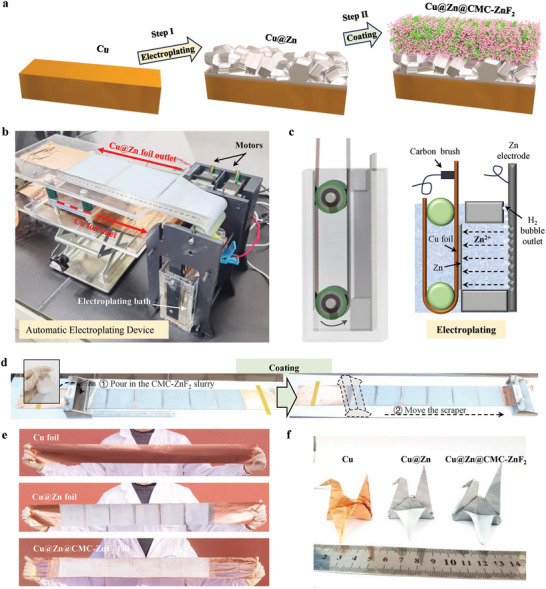
A large‐scale two‐step method based on electroplating and coating creating three‐in‐one zinc anodes. a) Schematic diagram of a three‐in‐one zinc anode by a two‐step method. b) A photo of an automatic electroplating device converting the Cu foil into the Cu@Zn foil. c) The 3D structure diagram of electroplating bath and schematic diagram of electroplating. d) Photos of the coating process. e) Photos of a Cu foil, a Cu@Zn foil and a Cu@Zn@CMC‐ZnF_2_ foil. f) Flexibility display of a Cu foil, a Cu@Zn foil and a Cu@Zn@CMC‐ZnF_2_ foil.

Herein, we propose a three‐in‐one zinc anode consisting of an inactive current collector, a zinc layer, and a protective layer to address the issues of uneven stripping and zinc dendrites. The three‐in‐one anode can be fabricated on a large scale and at low cost by a simple two‐step method involving electroplating and coating (**Figure** **1**a). In the first step, a Cu@Zn foil is obtained by depositing a dense zinc layer on top of the copper foil using an automatic electroplating device. Then in the second step, the blended slurry of sodium carboxymethyl cellulose and zinc fluoride (CMC‐ZnF_2_) in water is coated on the surface of Cu@Zn foil to obtain a three‐in‐one Cu@Zn@CMC‐ZnF_2_ foil. In this three‐in‐one anode, the copper foil acts as an inactive current collector below the zinc to ensure fast electron transfer and does not participate in battery reactions. When only pure zinc is used as the active collector, the local zinc may be completely stripped and the active region will gradually decrease from the in situ observation test of the transparent pouch cell. This phenomenon is aggravated as the size of the pouch cell increases. On the other hand, the CMC‐ZnF_2_ protective layer prevents direct contact between the glass fiber (GF) and zinc and improves the flux distribution of zinc ions. In the absence of a protective layer, point contacts between the non‐conducting glass fiber and the zinc surface are found and lead to uneven deposition of zinc and even the appearance of zinc dendrites. Consequently, the Cu@CMC‐ZnF_2_||Zn cell exhibits excellent cyclic reversibility up to 3000 cycles at 10 mA cm^−2^ and 2 mAh cm^−2^, achieving an exceptional average Coulombic efficiency of 99.94%, together with a remarkable cumulative deposition capacity of 6 Ah cm^−2^, which represents one of the best reported values. In contrast, the use of pure Cu foils under the same conditions results in only ≈ 500 cycles, an average Coulombic efficiency of 99.78%, and a deposition capacity limited to only 1 Ah cm^−2^. Subsequently, the full cell with the three‐in‐one Cu@Zn@CMC‐ZnF_2_ anode has a longer lifetime and better performance. It achieved more than 70% capacity retention after 15 000 cycles at a high current density of 10 A g^−1^, while the pure zinc anode fails after 242 cycles. In conclusion, the automatic electroplating device for preparing Cu@Zn anodes and the three‐in‐one zinc anode developed in this paper can greatly solve the difficulties faced by pure zinc anode. The relevant conclusions in this paper can provide basic insights for the research of practical thin zinc anode and accelerate the industrialization of practical zinc‐ion batteries.

## 2 Results

### 2.1 Automatically Electroplate the Cu Foil to the Cu@Zn Foil

In the two‐step method of Figure 1, the coating process of the second step can learn from the mature electrode coating process of lithium‐ion batteries. The electroplating of the first step has not yet been large‐scale preparation equipment. In the laboratory, a simple method for obtaining Cu@Zn foils is to assemble a copper foil, a zinc foil, and electrolyte into a battery with or without a separator. Then, apply current or voltage to move zinc ions from the zinc foil to the copper foil and finally obtain the galvanized copper foil. However, this simple method has the following problems, such as difficult to separate the deposited zinc from the separator, uneven deposition of zinc, restriction of deposition area, and waste of materials. Therefore, an automatic electroplating device is urgently needed to meet the needs of large‐scale preparation of Cu@Zn foils.

The automatic electroplating device we designed and manufactured successfully verifies the feasibility of automatic electroplating Cu@Zn foil. Figure 1b shows a photo of this device. A long and thin copper foil with a width of 10 cm is used as the deposition base, entering the automatic electroplating device on one side and coming out the other side after electroplating to become Cu@Zn foil. The movement of the entire device is achieved by two motors driving two driving wheels, which in turn drive three driven pulleys (Figure S1, Supporting Information). Through the curves of electroplating voltage, electroplating current and motor drive (Figure S2, Supporting Information), it can be observed that this device achieves continuous electroplating by cycling between electroplating and motor movement. The efficiency of this device primarily depends on the size of the plating area and the duration of each plating cycle. Figure 1c shows a 3D structure diagram and working principle diagram of the electroplating pool, which is one of the key components in this automatic electroplating device. Once a predetermined deposition capacity is reached, drivers drive driven pulleys to move up the right copper foil. Additionally, exhaust holes are designed to allow gas produced during electroplating to be discharged upward in order to enhance deposition uniformity. During electroplating, a large number of bubbles were observed on both sides of copper foil as well as on the zinc source side. A milky white CMC‐ZnF_2_ paste can be applied using a scraper for coating process on Cu@Zn foil (Figure 1d). Real photos showing Cu foil, Cu@Zn foil, and Cu@Zn@CMC‐ZnF_2_ foil are presented in Figure 1e. After undergoing this two‐step preparation process, flexibility is maintained without obvious loss (Figure 1f).

### 2.2 CMC‐ZnF_2_ Coating Enhancing Long‐Term Zinc Deposition/Stripping Reversibility

**Figure 2:**
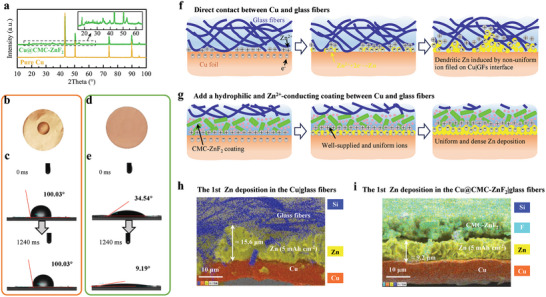
Zinc growth behavior on Cu foils with and without coating. a) XRD patterns. b) A wetting photo and c) contact angles of the Cu foil. d) A wetting photo and e) contact angles of the Cu@CMC‐ZnF_2_ foil. Schematic illustrations of electric and ionic distributions at f) the Cu|GFs interface and g) the Cu|CMC‐ZnF_2_ interface during Zn depositing. EDS images after the first Zn deposition in h) the Cu|GFs and i) the Cu@CMC‐ZnF_2_ for 5 mAh cm^−2^.

To illustrate the role of the CMC‐ZnF_2_ coating in regulating the ordered zinc deposition/stripping, the Cu@CMC‐ZnF_2_ foil was obtained by coating the copper foil with CMC‐ZnF_2_ slurry and drying it (Methods and Figure S3, Supporting Information). It is obviously observed from the X‐ray diffraction (XRD) patterns in **Figure** **2**a that a series of minor peaks are present at 2*θ* values ranging from 15° to 65° in the Cu@CMC‐ZnF_2_ sample, which is different from that of the pure Cu sample. Scanning electron microscope (SEM) images demonstrate that the surface of pure Cu foil is flatter than that of the Cu@CMC‐ZnF_2_ foil, which consists of agglomerated ZnF_2_ pellets and CMC prisms (Figure S4, Supporting Information). The morphology of the primeval CMC and ZnF_2_ powder can be seen in Figures S5 and S6, Supporting Information, respectively. Upon thorough stirring in water, cylindrical‐shaped CMC transforms into prismatic structures approximately ten microns long. The terminal face of each CMC prism is porous, which aids in trapping moisture (Figure S7, Supporting Information).

The CMC‐ZnF_2_ coating transforms the hydrophobic surface of the Cu foil into a superhydrophilic surface. As depicted in Figure 2b, when a 50 µL aqueous electrolyte is dropped onto a 19 mm diameter Cu foil, the droplet does not actively spread out. Precise measurement of the dynamic contact angle reveals that it remains constant at ≈100.03° from 0 to 1240 ms (Figure 2c). In contrast, the same electrolyte droplet on the Cu@CMC‐ZnF_2_ foil rapidly spreads out, confirming its hydrophilicity (Figure 2d). The contact angle between the electrolyte droplet and the surface of the Cu@CMC‐ZnF_2_ foil decreases from an initial value of 34.54° at 0 ms to 9.19° at 1240 ms (Figure 2e). When the contact angle of one material is less than 10°, it can be classified as superhydrophilic.

The CMC‐ZnF_2_ coating enhances the uniformity of ion and electron fields at the electrode/electrolyte interface by improving their distribution. As depicted in Figure 2f, zinc preferentially grows between the gaps of the glass fibers (GFs) when copper foil is directly in contact with GFs due to their large micro‐level pores within GF separator and nonconductive for ions and electrons themselves. This localized preferential deposition leads to non‐uniform electron field distribution, which intensifies with increasing deposition capacity and results in the formation of zinc dendrites. Therefore, utilizing pure Cu foil as a substrate and direct contact with nonconducting GFs for zinc deposition does not effectively address the issue of zinc dendrite growth.^[^ ^29^ ^]^ However, this problem is effectively mitigated by our coating approach, as shown in Figure 2g, where it prevents direct contact between Cu foil and GFs while ensuring a well‐supplied and homogeneous surface for zinc‐ion diffusion owing to both superhydrophilicity and ability to conduct zinc ions. Consequently, zinc ions can permeate through both GF separator and CMC‐ZnF_2_ layer resulting in a dense and uniform deposition on Cu substrate. Good initial deposition quality plays a crucial role in ensuring the cycle stability of batteries.^[^ ^36^ ^]^

The validation of the aforementioned process was conducted using Energy Dispersive Spectrometer (EDS) diagrams of the sample cross‐section, which illustrate the initial deposition of zinc in both Cu|GFs and Cu@CMC‐ZnF_2_ systems. As shown in Figure 2h and Figure S8, Supporting Information, when direct contact is established between the Cu foil and GFs, the distribution of zinc deposition becomes highly uneven, with certain areas penetrating into gaps within the GF separator. For a zinc deposition of 5 mAh cm^−2^, the maximum height can reach ≈15.6 µm. In contrast, upon incorporating a protective layer of CMC‐ZnF_2_, zinc deposition becomes remarkably uniform and thinner (≈9.2 µm), confined exclusively to the region between the Cu layer and the CMC‐ZnF_2_ layer (Figure 2i). ​Furthermore, if hydrophilic CMC‐ZnF_2_ coating is substituted with hydrophobic PVDF‐ZnF_2_ coating, significant looseness and non‐uniformity are observed in deposited zinc despite its presence between the coating and the Cu layer, too (Figure S9, Supporting Information). Compared to the CMC‐ZnF_2_ coating, the PVDF‐ZnF_2_ appears excessively dense (Figure S10, Supporting Information), while hydrophobicity of PVDF itself does not facilitate efficient transport of zinc ions (Figure S11, Supporting Information). Consequently, there is scarcity of zinc ions on Cu foil surface leading to considerable reduction in density and homogeneity during zinc deposition (Figure S12, Supporting Information).

The surface morphology of Zn deposited beneath the coating was observed by removing localized coatings using a tweezer. Figure S13, Supporting Information shows EDS images revealing a dense yellow Zn layer on top of an orange Cu foil and below a cyan CMC‐ZnF_2_ layer after the initial deposition at 5 mAh cm^−2^. Further stripping causes Zn to disappear from the Cu@Zn@CMC‐ZnF_2_ structure. Overall, the CMC‐ZnF_2_ coating effectively controls zinc growth region for uniform, dense growth and sufficient stripping between the zinc‐ion conductor (CMC‐ZnF_2_ layer) and electronic conductor (Cu foil). The deposition morphology of Zn in Cu@CMC‐ZnF_2_ after the 20th deposition with a deposition capacity of 5 mAh cm^−2^ is illustrated in Figure S14, Supporting Information. It can be seen that the height of the zinc layer increases from 9.2 µm during initial deposition (Figure 2i) to 16.6 µm after the 20th deposition, indicating decreased density over time. SEM images demonstrate that Zn deposition remains confined between the coating and Cu foil, suggesting that restricting the deposition region strategy remains effective throughout cycles. Additionally, EDS mappings reveal a distinct boundary between sulfur element and Zn layers, implying that this coating may effectively inhibit sulfur penetration into deposited Zn.

**Figure 3:**
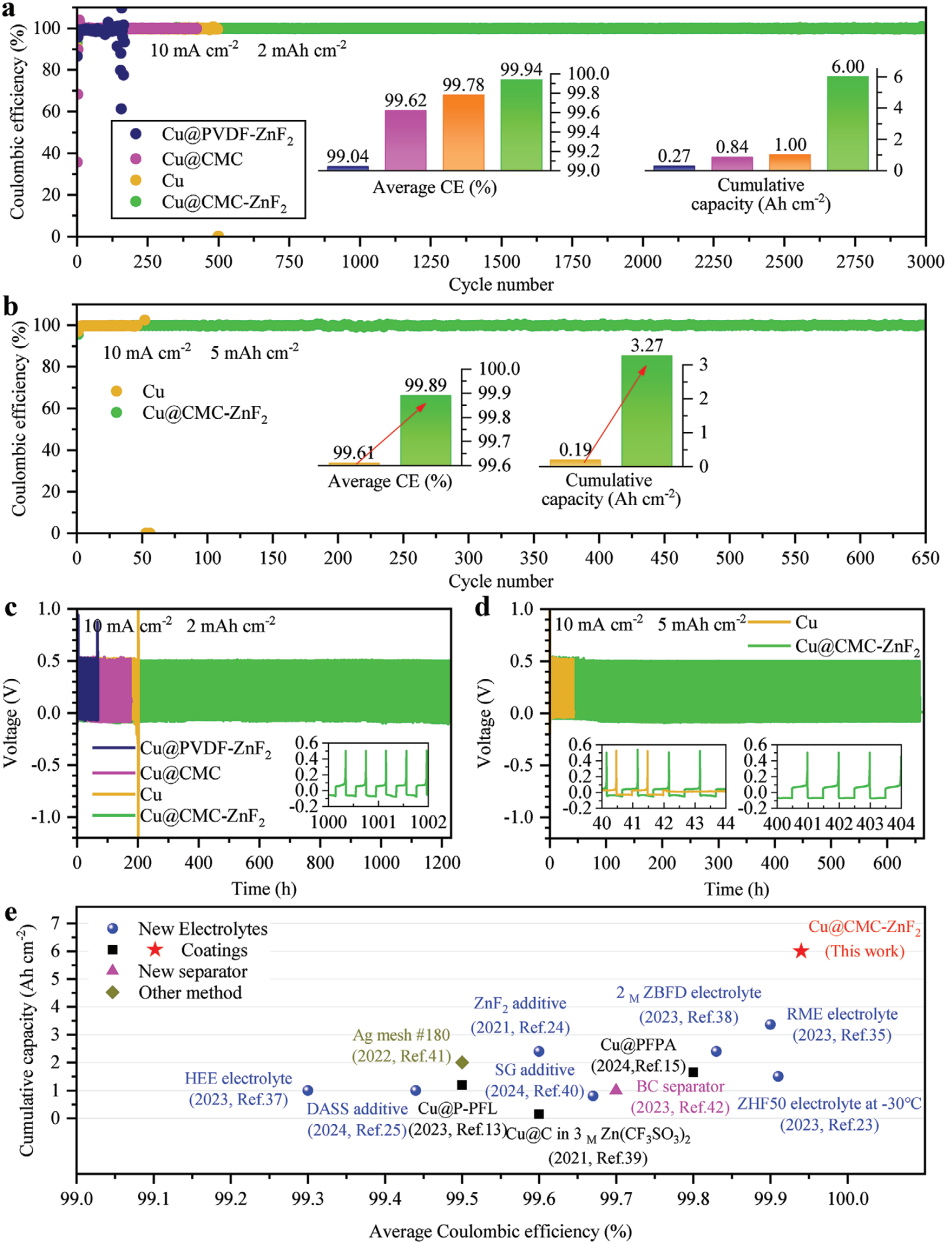
Electrochemical properties of Zn||Cu cells. a) Cycling performances of Zn||Cu cells using various Cu foils at 10 mA cm^−2^ and 2 mAh cm^−2^. b) Cycling performances of Zn||Cu cells using a Cu foil and a Cu@CMC‐ZnF_2_ foil at 10 mA cm^−2^ and 5 mAh cm^−2^. The voltage curves of Zn||Cu cells at 10 mA cm^−2^ c) for 2 mAh cm^−2^ and d) for 5 mAh cm^−2^. e) Comparison of average Coulombic efficiencies and cumulative capacities of various Zn||Cu/Ag/Ti cells in recent years.

The lifetimes and Coulombic efficiencies of Zn||Cu half cells serve as crucial indicators of the long‐term reversibility of Zn deposition/stripping on the Cu side. First, four different Cu electrodes are employed in Zn||Cu half cells: a pure Cu foil, a Cu@CMC foil, a Cu@CMC‐ZnF_2_ foil and a Cu@PVDF‐ZnF_2_ foil (**Figure** **3**a). The voltage curves of these Zn||Cu half‐cells at different cycles are shown in Figure S15, Supporting Information. Among these four electrodes, the reversibility of zinc deposition/stripping is superior for the Cu@CMC‐ZnF_2_ electrode compared to others. The cycling stability of the Cu@CMC‐ZnF_2_ electrode is exceptional up to 3000 cycles at 10 mA cm^−2^ and 2 mAh cm^−2^, exhibiting an impressive average Coulombic efficiency of 99.94% and a substantial cumulative capacity reaching 6 Ah cm^−2^. In contrast, under identical conditions, the pure Cu electrode only achieves limited cycling stability with less than 500 cycles along with a restricted average Coulombic efficiency of 99.78% and a limited cumulative capacity of 1 Ah cm^−2^. The reason for failure observed for the Cu foil can be seen in Figure 3c where there is significant voltage polarization increase after 450 cycles. Moreover, both the Cu@CMC foil and the Cu@PVDF‐ZnF_2_ foil exhibit worse performance than the Cu@CMC‐ZnF_2_ foil and the Cu foil. Thus achieving excellent performance requires utilizing synergistic effects between ZnF_2_ and CMC while presenting challenges when only use CMC or combine PVDF with ZnF_2_.

When the deposition capacity is increased from 2 to 5 mAh cm^−2^ (Figure 3b), the Cu@CMC‐ZnF_2_ electrode exhibits remarkable cycling stability, sustaining over 650 cycles with an average Coulombic efficiency of 99.89% and a cumulative capacity of 3.27 Ah cm^−2^. In contrast, the pure Cu foil can only endure less than 50 cycles, displaying lower average Coulombic efficiency (99.61%) and cumulative capacity (0.19 Ah cm^−2^). Notably, as depicted in Figure 3d and Figure S16, Supporting Information, an internal short circuit eventually impedes the charge voltage of the Zn||pure Cu cell from reaching the cutoff voltage of 0.5 V.

The average Coulombic efficiency and cumulative capacity, as shown in Figure 3e, are employed as comparative indicators to evaluate the performance of Zn||Cu/Ag/Ti batteries with different electrodes and electrolytes reported in recent years, including RME electrolyte,^[^ ^35^ ^]^ HEE electrolyte,^[^ ^37^ ^]^ 2 _M_ ZBFD electrolyte,^[^ ^38^ ^]^ ZHF50 electrolyte at −30 °C,^[^ ^23^ ^]^ Cu@C in 3 _M_ Zn(CF_3_SO_3_)_2_,^[^ ^39^ ^]^ ZnF_2_ additive,^[^ ^24^ ^]^ DASS additive,^[^ ^25^ ^]^ SG (sodium alginate) additive,^[^ ^40^ ^]^ Ag mesh #180,^[^ ^41^ ^]^ Cu@PFPA,^[^ ^15^ ^]^ Cu@P‐PFL,^[^ ^13^ ^]^ and BC separator.^[^ ^42^ ^]^ It is worth noting that the morphology of deposited zinc significantly influences the electrode performance. Due to the dense and uniform deposition of zinc observed in the Cu@CMC‐ZnF_2_ electrodes, it exhibits certain advantages over other investigated systems.

### 2.3 Growth Behavior of Zinc at Different Capacities and Cycles

**Figure 4:**
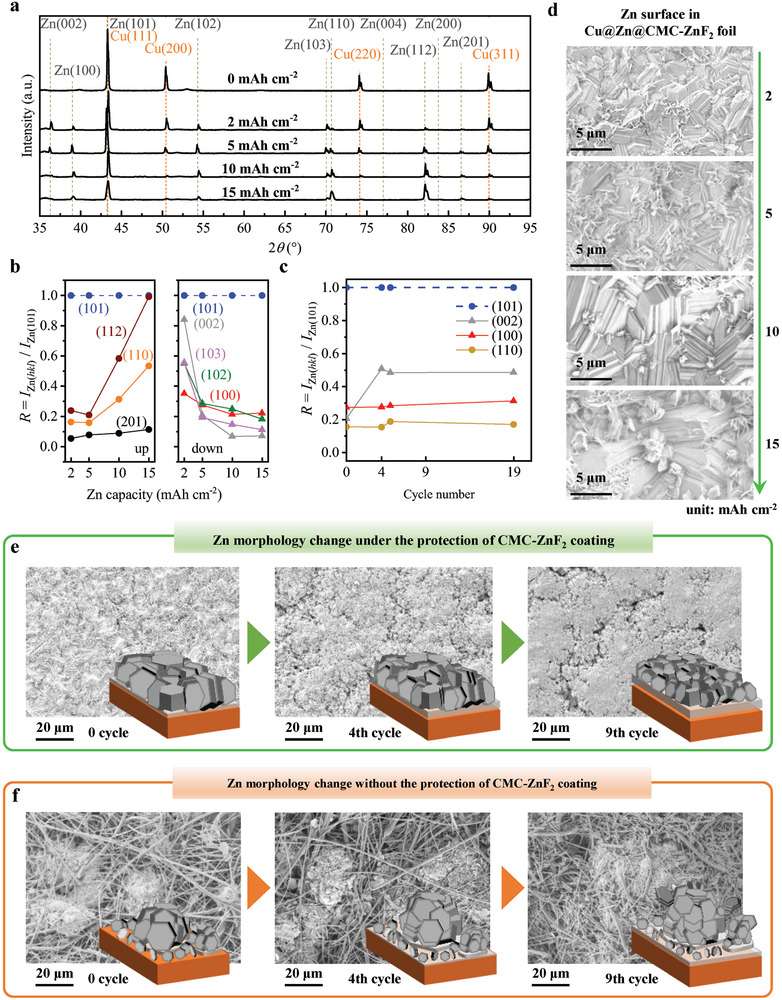
XRD patterns and morphologies of zinc growth at different deposition capacities and cycle numbers. a) XRD patterns of Cu@Zn@CMC‐ZnF_2_ foils under various deposition capacities of Zn. Changes of the relative texture coefficients for different crystalline phases as b) the increasing Zn deposition capacity and c) the increasing cycle number under 5 mAh cm^−2^. d) ​Morphology of Zn deposited in Cu@CMC‐ZnF_2_ foils at various Zn deposition capacities. SEM images and 3D schematics of e) uniform Zn deposition in Cu@CMC‐ZnF_2_ foils and f) dendritic deposition of Zn on Cu foils with cycle number increases at 10 mA cm^−2^ and 5 mAh cm^−2^.

It is imperative to comprehend the underlying law governing the alteration of number and size of zinc crystal planes in the Cu@CMC‐ZnF_2_ foil as zinc grows. XRD patterns were measured for Cu@Zn@CMC‐ZnF_2_ foils with various deposition capacities (0, 2, 5, 10, and 15 mAh cm^−2^), as shown in **Figure** **4**a. The intensities of Cu(111), Cu(200), Cu(220), and Cu(311) peaks gradually decline with increasing Zn deposition capacity. Referenced 2*θ* values of these Cu crystal planes can be found in Table S2, Supporting Information. Zn‐related peaks emerge in samples with deposition capacities of 2, 5, 10 and 15 mAh cm^−2^. The observed trend in the variation of the Zn peak suggests some level of selectivity during the growth of the Zn crystal plane.

To determine the growth trend of each Zn crystal direction, it is assumed that the intensity ratio between Cu(111) peak and Cu(200) peak remains constant. The intensity of Cu(111), which overlaps with Zn(101), can be calculated using the Cu(200) peak. Subsequently, the intensity of Zn(101) can be determined based on the total peak height at 2*θ*≈43.2°. Based on these intensity values, it can be concluded that Zn(101) is the dominant crystal face in the deposited Zn layer. The relative texture coefficient *R* for other Zn crystal planes can be calculated using the formula *R* = *I*_Zn(_ *_hkl_*_)_/*I*_Zn(101)_, and results are shown in Figure 4b. The relative texture coefficients of Zn(004) and Zn(200) are ≈0; hence they are not shown in Figure 4b. Other crystal plane growth trends can be broadly categorized into two groups: up and down. Specifically, relative texture coefficients of Zn(112), Zn(110), and Zn(201) crystal planes exhibit an uptrend with increasing deposition capacity. Referenced 2*θ* values of these Zn crystal planes and their angles between their normal vectors and the substrate can be found in Table S3, Supporting Information. The Zn(112) crystal plane exhibits the largest increase, with the *R* value increasing from ≈0.24 at 2 mAh cm^−2^ to ≈0.99 at 15 mAh cm^−2^. In contrast, the relative texture coefficients of Zn(002), Zn(103), Zn(102), and Zn(100) crystal planes show a down trend. The Zn(002) crystal plane, characterized by a normal vector perpendicular to the substrate, experiences the most significant reduction in *R* value, decreasing from ≈0.84 at 2 mAh cm^−2^ to ≈0.07 at 15 mAh cm^−2^. Therefore, during the initial stage of zinc deposition in the Cu@CMC‐ZnF_2_ foil, a substantial amount of Zn(002) vertical crystal planes is formed. As deposition progresses, there is a gradual decrease in the quantity of Zn(002) vertical crystal planes while an increase is observed in Zn(112) inclined crystal planes.

XRD tests were conducted on Cu@Zn@CMC‐ZnF_2_ at various cycle numbers (0, 4, 9, and 19) (Figure S17, Supporting Information). For example, a cycle number of 4 indicates four deposition/stripping cycles followed by one deposition. The deposition capacity is fixed at 5 mAh cm^−2^, and cutoff voltage of the stripping process is set at 0.5 V. The relative texture coefficients for different Zn crystal planes are shown in Figure 4c. The *R* value for Zn(002) exhibits an increasing trend, rising rapidly from 0.2 during the first deposition to 0.51 during the fifth deposition (4 cycles), and then stabilizing at ≈0.5 during cycles 4 to 19. This result suggests that after multiple depositions, the crystal orientation in the Cu@Zn@CMC‐ZnF_2_ foil remains stable.

The SEM images in Figure 4d depict the surface morphology of the Cu@Zn@CMC‐ZnF_2_ electrode after ultrasonic removal of the CMC‐ZnF_2_ layer using water. Most crystal planes exhibit inclination, consistent with the XRD results. Even at a deposition capacity of 15 mAh cm^−2^, the sedimentary structure of zinc remains compact. With an increase in Zn deposition capacity, there is a significant enlargement in crystalline size, indicating that dominant homogeneous epitaxial growth during zinc growth. Simultaneously, spherical zinc‐deposited seeds form at interfaces between different textures of zinc, suggesting continuous formation of new nuclei. Figure 4e illustrates the Cu@CMC‐ZnF_2_ surface after 0, 4, and 9 cycles where the ultrasonic removal of the CMC‐ZnF_2_ layer facilitates our observation. The grain size of deposited zinc decreases noticeably as the number of cycles increases. This trend is depicted vividly in the 3D schematics on the right side of Figure 4e, in which CMC‐ZnF_2_ layer is hidden. The repeated deposition/stripping process at texture interfaces leads to an increasing nucleation, resulting in a gradual reduction in size and densification of zinc deposition. The deposition of Zn on the pure Cu foil shown in Figure 4f is not uniform. Within GFs on the pure Cu foil, zinc dendrites infiltrate and continue to grow during cycling dissolution and deposition. The presence of non‐conductive GFs disrupts the uniformity of zinc ion fields. Zinc dendrites cause uneven electron distribution. Consequently, new nucleation becomes non‐uniform because of uneven ion fields and electron fields, resulting in continuous deterioration along with cycle increase.

### 2.4 Solve the Problem of Decreasing Active Area of the Active Current Collector

**Figure 5:**
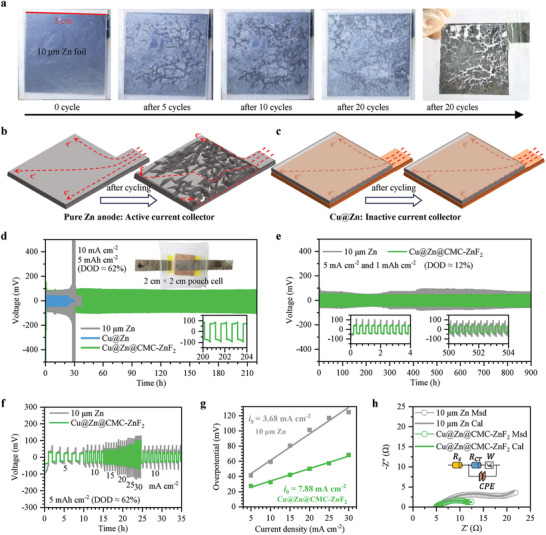
The bad effect of pure Zn as an active current collector on the lifetime of the pouch cell. a) The decrease of the active area of the pure Zn electrode with the increase of the cycle number observed in a transparent pouch cell. Schematic illustrations of electron transport on b) an active current collector of pure Zn and c) an inactive current collector of Cu before and after cycling. d) Galvanostatic long‐cycle data of symmetrical pouch cells of 2 × 2 cm using different electrodes at 10 mA cm^−2^ and 5 mAh cm^−2^. e) Galvanostatic long‐cycle data of symmetrical coin cells using 10 µm Zn electrodes and Cu@Zn@CMC‐ZnF_2_ electrodes at 5 mA cm^−2^ and 1 mAh cm^−2^. f) Rate performances of symmetrical coin cells using 10 µm Zn electrodes and Cu@Zn@CMC‐ZnF_2_ electrodes. g) The exchange current densities. h) EIS curves of a Ti mesh||Zn cell and a Ti mesh||Cu@Zn@CMC‐ZnF_2_ cell.

The deposited zinc capacity in the Cu@Zn@CMC‐ZnF_2_ foil is designed to match the theoretical capacity of ≈8.05 mAh cm^−2^ for the 10 µm zinc foil used (Table S3, Supporting Information). A transparent 10 µm Zn symmetric pouch cell was assembled using a transparent sealed bag to visualize the decrease in active area of the pure zinc electrode. This pouch cell had an effective active area of 5 × 5 cm, used a GF separator, and employed an electrolyte with a density of 50 µL cm^−2^. As shown in **Figure** **5**a, optical photos depict changes in the zinc electrode (stripping side first) with increasing cycle number under conditions of 10 mA cm^−2^ and 5 mAh cm^−2^. The pure zinc electrode can be referred to as an active current collector, as it not only participates as an active material, but also acts as an electron collector during reactions involving the Zn and Zn^2+^ transitions. Uneven stripping and deposition may cause local areas to experience complete stripping that becomes more severe with increasing cycle number when using thin pure zinc as an active current collector, leading to gradual reduction in active area that affects rapid electron transport (Figure 5b). However, this problem does not occur with Cu@Zn electrodes formed by depositing zinc on copper foil since copper serves solely as an inactive current collector without participating in electrode reactions (Figure 5c).

Galvanostatic long‐cycle testing for Zn||Zn symmetric batteries can detect the ability of various electrodes to withstand stripping/deposition and deposition/stripping over extended periods. To minimize interference from the stainless steel shell and highlight the crucial role of the inactive current collector, we initially assembled 2 × 2 cm symmetric pouch cells to evaluate their galvanostatic long cycle performance at a high current density of 10 mA cm^−2^ and a high areal capacity of 5 mAh cm^−2^ (DOD≈62%). The test results and photo of this pouch cell using Cu@Zn@CMC‐ZnF_2_ electrodes are shown in Figure 5d. The Cu@Zn@CMC‐ZnF_2_ symmetric battery exhibits a lifetime exceeding 210 h, significantly outperforming both the Cu@Zn symmetric battery (≈25 h) and the 10 µm Zn symmetric battery (≈30 h). This demonstrates the effectiveness of integrating zinc with an inactive current collector and employing a CMC‐ZnF_2_ protective layer for the Cu@Zn@CMC‐ZnF_2_ electrode. In terms of coin cells, as depicted in Figure 5e, the Cu@Zn@CMC‐ZnF_2_ symmetric battery displays lower polarization and more stable voltage compared to the 10 µm Zn symmetric battery.

As shown in Figure 5f, the rate performances of symmetric cells using 10 µm Zn electrodes and Cu@Zn@CMC‐ZnF_2_ electrodes were tested, from 5 to 25 mA cm^−2^ at a constant capacity of 5 mAh cm^−2^. The exchange current densities^[^ ^29^, ^43^ ^]^ of symmetric cells using 10 µm Zn electrodes and Cu@Zn@CMC‐ZnF_2_ electrodes are 3.68 and 7.88 mA cm^−2^, respectively (Figure 5g). The charge transfer impedance of 10 µm Zn foil and Cu@Zn@CMC‐ZnF_2_ foil were accurately compared using a three‐electrode system for electrochemical impedance spectroscopy (EIS) testing. This system comprised an Ag/AgCl reference electrode, a 2 × 2 cm Ti mesh counter‐electrode, a 1 cm^2^ working electrode and the aqueous electrolyte (Figure S18, Supporting Information). The results are presented in Figure 5h. Their internal resistance (*R*_s_) values of the Zn foil and the Cu@Zn@CMC‐ZnF_2_ foil were 5.35 and 4.91 Ω, respectively. However, there was a significant difference in the charge transfer impedance (*R*_CT_), with values of 7.95 Ω for the Cu@Zn@CMC‐ZnF_2_ foil and 14.08 Ω for the Zn foil, indicating that the obstruction of charge transfer on the Cu@Zn@CMC‐ZnF_2_ electrode is lower. The above results strongly prove that the Cu@Zn@CMC‐ZnF_2_ symmetric battery can perform better when the Cu@Zn@CMC‐ZnF_2_ and 10 µm Zn electrodes have the same capacity.

The above conclusions are derived from comparisons conducted under identical DOD conditions. It should be noted, however, that the DOD calculation cannot account for the weight of the inactive collector layer and the coating. It can be calculated that the mass ratio of copper, zinc, and coating in the three‐in‐one zinc foil fabricated using 10 µm Cu foil is ≈49.8%, 44.3%, and 5.9%, respectively (Table S4, Supporting Information). On one hand, both copper and coating contribute to an increase in the mass of inactive components within the battery, consequently reducing its energy density. On the other hand, their presence ensures structural integrity of the thin electrode while enhancing battery performance to improve battery energy density and lifetime. In a sense, these advantageous effects achieved through the three‐in‐one foils come at the expense of sacrificing the content of active matter within the said electrodes. To increase active substance proportion within the anode, potential strategies include reducing copper foil thickness, increasing zinc layer capacity, and using microporous copper foils.

### 2.5 Excellent Performance of Full Batteries Using Three‐in‐One Anodes

**Figure 6:**
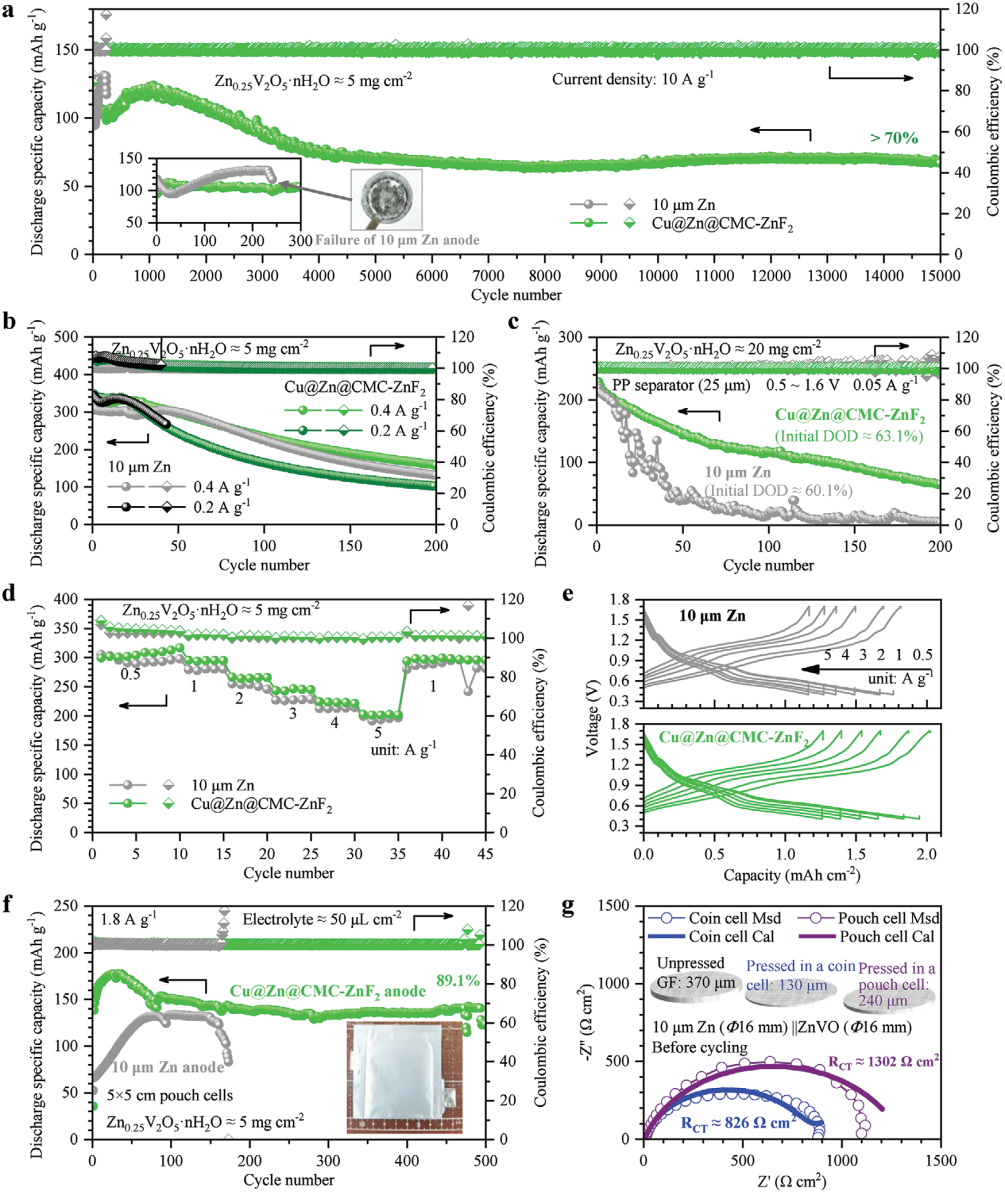
Performances of Zn||ZnVO cells. Comparison of long‐term performance of Zn|GF|ZnVO coin cells using 10 µm Zn anodes and Cu@Zn@CMC‐ZnF_2_ anodes at a) a high current density of 10 A g^−1^, b) low current densities of 0.4 and 0.2 A g^−1^. c) Comparison of long‐term performance of Zn|PP|ZnVO coin cells using a 10 µm Zn anode and a Cu@Zn@CMC‐ZnF_2_ anode at a low current density of 0.05 A g^−1^. Comparisons of d) rate performances and e) voltage curves of Zn|GF|ZnVO cells using a 10 µm Zn anode and a Cu@Zn@CMC‐ZnF_2_ anode. f) Long‐term performance of pouch cells of 5 × 5 cm and a photo of the Cu@Zn@CMC‐ZnF_2_||ZnVO pouch cell of 5 × 5 cm (insert image). g) EIS curves of the 10 µm Zn||ZnVO coin cell and pouch cell, and the thickness of GF separators in three states (insert images).

The cathode material chosen for validating the performance of Cu@Zn@CMC‐ZnF_2_ in a full battery system was ZnVO, which exhibited exceptional conductivity (Figure S19, Supporting Information). As depicted in **Figure** **6**a, the Cu@Zn@CMC‐ZnF_2_ anode demonstrates excellent performance in a full cell configuration with a ZnVO load of 5 mg cm^−2^, a high current of 10 A g^−1^, and a voltage range of ≈0.4–1.7 V. The initial discharge specific capacity of the cell reaches 94.97 mAh g^−1^. ​The discharge specific capacity remains at 67.68 mAh g^−1^ even after 15 000 cycles. Then this battery has a capacity retention of over 70% after 15 000 cycles. In contrast, when using a 10 µm Zn anode, the full battery failed after ≈242 cycles due to internal short circuiting (Figure S20, Supporting Information). The charge and discharge curves of the two cells in different cycles are depicted in Figure S21, Supporting Information, revealing enhanced cyclic stability for the cell employing the Cu@Zn@CMC‐ZnF_2_ anode. Upon peeling off the failed zinc anode, it was observed that the circular effective region had completely stripped (insert image in Figure 6a). As shown in Figure 6b, at a low current of 0.4 A g^−1^, the Zn|GF|ZnVO cell using a Cu@Zn@CMC‐ZnF_2_ anode also outperforms that using a 10 µm Zn anode, and the discharge specific capacity after 200 cycles is 158.75 and 131.83 mAh g^−1^, respectively. By further reducing the current to 0.2 A g^−1^, the initial discharge specific capacity of the cell is further increased to 347.44 and 343.56 mAh g^−1^ for using a Cu@Zn@CMC‐ZnF_2_ anode and a 10 µm Zn, respectively. At 0.2 A g^−1^, the cell using a 10 µm Zn anode shows shorter life, significantly overcharging on the 41^st^ cycle and dying after the 43^rd^ cycle. In general, Zn|GF|ZnVO coin cells using Cu@Zn@CMC‐ZnF_2_ anodes have better long‐term performance than those using 10 µm Zn anodes. Moreover, the Zn||ZnVO cells exhibit poorer cyclic stability at lower current densities, which could be attributed to the inadequate stability of the vanadium‐based cathode under low current densities. At a low current density, the intercalation reaction of Zn^2+^ and H^+^ is more complete, and the cathode structure is more likely to be damaged, resulting in irreversible capacity loss.^[^ ^44^ ^]^ Previous research has demonstrated that the dissolution of vanadium‐based cathode materials can be mitigated by employing modified separators ^[^ ^19^ ^]^ and novel electrolytes, both of which directly interact with the cathode. These include a polyacrylonitrile (PAN)‐based separator,^[^ ^19^ ^]^ a Zn‐Nafion separator,^[^ ^45^ ^]^ a separator combining polyvinylidene difluoride with polydopamine (PVDF@PDA),^[^ ^46^ ^]^ an aqueous electrolyte consisting of 2 _M_ Zn(OTF)_2_ and 8 _M_ LiOTF,^[^ ^47^ ^]^ and a quasi‐solid electrolyte comprising a high solid content of CaSO_4_·2H_2_O and a low liquid content of ZnSO_4_.^[^ ^48^ ^]^

To increase the energy density, we tried to use a thinner separator and a higher load cathode. In Figure 6c, the GF separator (≈370 µm) is replaced by the hydrophilic PP separator (≈25 µm), and the ZnVO electrode prepared by the coating method is replaced with a self‐supporting ZnVO cathode, which has a high load of ≈20 mg cm^−2^. When paired with the Cu@Zn@CMC‐ZnF_2_ anode, the cell can work normally under a low current density of 0.05 A g^−1^ and a voltage range of ≈0.5–1.6 V. The discharge specific capacity of the cell in the first cycle reaches 228.80 mAh g^−1^, the discharge capacity exceeds 5 mAh cm^−2^, and the DOD is ≈63.1%. After 100 cycles, the capacity retention is ≈51.38%. In contrast, the initial discharge specific capacity and DOD of the cell using a 10 µm Zn anode are 218.77 mAh g^−1^ and ≈60.1%, respectively. Capacity retention is as low as ≈8.2% after 100 cycles. Therefore, this three‐in‐one zinc anode is promising in the application scenarios of a high DOD and thin separator.

In the rate test depicted in Figure 6d, the specific discharge capacity of the Cu@Zn@CMC‐ZnF_2_ anode can reach up to 299.38, 295.51, 266.61, 243.61, 224.28, and 202.49 mAh g^−1^ at current densities of 0.5, 1, 2, 3, 4, and 5 A g^−1^, respectively. Under identical variations in current density, the performance of a 10 µm Zn anode is slightly inferior with discharge specific capacities reaching such as 305.25, 279.90, 255.42, 227.12, 212.93, and 199.41 mAh g^−1^, respectively. Voltage‐capacity curves of these two batteries are shown in Figure 6e, the battery using Cu@Zn@CMC‐ZnF_2_ anode has a higher capacity than a battery using 10 µm Zn anode at different current densities. Therefore, the Cu@Zn@CMC‐ZnF_2_ composite exhibits superior performance at large unit area currents and long cycles compared to the use of pure zinc as the anode material alone.

The applicability of the new‐type anode in pouch cells was tested by assembling pouch cells of 2 × 2 and 5 × 5 cm. Figure S22, Supporting Information illustrates the cycle performance obtained from a 2 × 2 cm pouch cell using this three‐in‐one anode under a current of 2 A g^−1^. The initial discharge specific capacity is measured at 93.59 mAh g^−1^, while the discharge specific capacity for the second cycle is recorded as 138.37 mAh g^−1^. After undergoing 500 cycles, the discharge specific capacity reaches 157.88 mAh g^−1^, resulting in a calculated capacity retention of ≈114% based on 138.37 mAh g^−1^. Upon reaching its thousandth cycle, the discharge specific capacity drops to 104.92 mAh g^−1^ with a corresponding capacity retention of 75.8%. As shown in Figure 6f, the larger 5 × 5 cm pouch cell employing this three‐in‐one anode exhibits a discharge specific capacity of 138.43 mAh g^−1^ during the second cycle under a current of 1.8 A g^−1^. After undergoing 500 cycles, the discharge specific capacity remains at 123.40 mAh g^−1^, resulting in an approximate capacity retention of 89.1% based on 138.43 mAh g^−1^. In contrast, when using a 10 µm Zn anode in a pouch cell measuring 5 × 5 cm, fewer cycles are achieved due to micro short circuits occurring within the battery. After only 173 cycles, significant overcharge occurs and develops into a complete short circuit, causing the voltage to drop to zero (Figure S23, Supporting Information). Furthermore, compared to the cell utilizing a three‐in‐one anode, the pouch cell with a 10 µm Zn anode exhibit inferior performance in terms of discharge specific capacity. The latter reaches its maximum discharge specific capacity of 132.27 mAh g^−1^ on the 82^nd^ cycle, which is fewer than that of 177.49 mAh g^−1^ achieved on the 33^rd^ cycle by the former. Therefore, the Cu@Zn@CMC‐ZnF_2_ anode can be effectively used in pouch cells, and it shows good performance at large current densities.

Under identical test conditions, the performance of coin cells in zinc‐ion batteries often exhibits significant superiority over pouch cells, as reported in previous literature.^[^ ^49^, ^50^ ^]^ In this paper, the coin cell reaches 266.61 mAh g^−1^ at 2 A g^−1^ (Figure 6d), while the pouch cell only reaches 159.57 mAh g^−1^ at 2 A g^−1^ during the 21^st^ cycle (Figure S22, Supporting Information). We believe that a key factor causing this phenomenon is the change in thickness of the thick and elastic GF separator after battery pressing. As shown in Figure S24, Supporting Information, vacuum compression (>20 s, <−95 kPa) is applied to the pouch cell, while mechanical pressure (>50 kg cm^2^) is used for the coin cell. The thickness of the GF separator under the press state is calculated by subtracting the thickness of each battery component after pressing from the total thickness of the battery. The unpressed GF separator has a measured thickness of ≈370 µm (Figure S25, Supporting Information). After pressing, the thickness of GF separators in coin cells with and without electrolyte are calculated to be 130 and 120 µm, respectively (Table S5, Supporting Information), whereas for pouch cells they are calculated to be 240 and 260 µm, respectively (Table S6, Supporting Information). Figure 6g visually illustrates differences of GF thicknesses in these three states (unpressed state, pressed state with electrolyte in a coin cell, pressed state with electrolyte in a pouch cell). It can be clearly observed that after pressing, there is a significant reduction in GF separator thicknesses and that in a coin cell it is about half as thin compared to that in a pouch cell. Moreover, after pressing, the thickness of ZnVO cathode in a coin cell also becomes thinner than that in a pouch cell (Tables S5 and S6, Supporting Information). Figure 6g displays the EIS results of coin and pouch cells assembled with electrodes of 16 mm diameter (Figure S26, Supporting Information). The used fitting circuit is the same as that in Figure 5h. The coin cell exhibits lower internal resistance (Rs ≈ 1.95 Ω cm^2^) and charge transfer impedance (R_CT_ ≈ 826 Ω cm^2^) compared to the pouch cell (Rs ≈ 3.07 Ω cm^2^ and R_CT_ ≈ 1302 Ω cm^2^). Therefore, the superior performance of coin cells compared to pouch cells in zinc‐ion batteries can be attributed to variations in the compression level of the thick GF separator resulting from the pressing process. The application of pressure on zinc‐ion pouch batteries with a thick separator during assembly or testing should be taken into consideration.

## 3 Conclusion

This work presented a three‐in‐one thin zinc anode fabricated by a scalable two‐step method. An automatic electroplating device was developed to realize the continuous preparation of Cu@Zn foils. Additionally, well‐designed CMC‐ZnF_2_ coating effectively prevents direct contact between zinc and glass fibers and improves zinc/zinc ion contact. The synergistic effect of hydrophilic CMC and ZnF_2_ in the coating facilitates uniform and well‐supplied zinc ions on the surface, resulting in dense and uniform zinc when depositing. Compared to pure Cu||Zn cells, Cu@CMC‐ZnF_2_||Zn cells exhibit superior cycling stability (3000 cycles) with an average Coulombic efficiency of 99.94% and a high cumulative capacity of 6 Ah cm^−2^ at 10 mA cm^−2^ and 2 mAh cm^−2^. Furthermore, we elucidate the regulatory mechanisms underlying zinc growth by comprehensively analyzing the coating properties and deposition locations. Additionally, we investigate the zinc growth properties under varying deposition capacities and cycle numbers while being protected by the CMC‐ZnF_2_ coating. The full batteries assembled with Cu@Zn@CMC‐ZnF_2_ anodes and ZnVO cathodes exhibit superior longevity and performance compared to those using 10 µm Zn foil, even when the theoretical capacity of the anodes is the same. The cycle number of the full cell equipped with this new‐type anode reaches up to 15 000 cycles while maintaining a high capacity retention over 70%. Furthermore, in a pouch cell measuring 5 × 5 cm area size, it demonstrates excellent capacity retention (89.1%) after undergoing 500 cycles at a loading of 5 mg cm^−2^ and high current density of 1.8 A g^−1^. These results are highly competitive compared to previously reported literature findings, thus accelerating the development of practical thin zinc anodes.

## Conflict of Interest

The authors declare no conflict of interest.

## Supporting information

Supporting Information

## Data Availability

The data that support the findings of this study are available from the corresponding author upon reasonable request.
