# Supplementary material for: Three‐in‐One Zinc Anodes Created by a Large‐scale Two‐Step Method Achieving Excellent Long‐Term Cyclic Reversibility and Thin Electrode Integrity
Source: Adv Sci (Weinh). 2024 May 20;11(28):2401575. doi: 10.1002/advs.202401575 (PMC11267265; doi:10.1002/advs.202401575)
Supplement: Supplementary file 1 — Supporting Information [file ADVS-11-2401575-s001.docx]

**Supporting Information for Three-in-one Zinc Anodes Created by A Large-scale Two-step Method Achieving Excellent Long-term Cyclic Reversibility and Thin Electrode Integrity**

Hongfei Lu^1^, Di Zhang^1^, Zhenjie Zhu^1^, Nawei Lyu^1^, Xin Jiang^1^, Chenxu Duan^1^, Yi Qin^1^, Xinyao Yuan^1^, Yang Jin^1*^

Dr. H. Lu, Mr. D. Zhang, Mr. Z. Zhu, Dr. N. Lyu, Dr. X. Jiang, Mr. C. Duan, Mr. Y. Qin, Miss X. Yuan, Prof. Y. Jin (Corresponding author)

Research Center of Grid Energy Storage and Battery Application, School of Electrical and Information Engineering, Zhengzhou University, Zhengzhou, Henan 450001, China

E-mail: luhfff@126.com, zhangdi3512@163.com, zhuzhenjie@gs.zzu.edu.cn, naweilyu@zzu.edu.cn, jiangxin@zzu.edu.cn, dcxchenxu@163.com, yiqin@gs.zzu.edu.cn, yuanxinyao8782@163.com, yangjin@zzu.edu.cn

1. Experimental Section

***Materials*:** Glass fiber separators (GF/C, Diameter 110 mm, 100 Circles, No.1822-110) were bought from Whatman. 3501 PP separators were bought from Celgard. V_2_O_5_ powder (AR) was bought from Enox Reagent. ZnSO_4_·7H_2_O (AR), ethyl alcohol (AR), acetone (AR), zinc acetate dihydrate (C_4_H_6_O_4_Zn·2H_2_O, AR) and Zn foil (200 μm thick, 99.9%) were bought from Sinopharm Chemical Reagent. 10 μm and 30 μm Zn foils were bought from Haiyuan Technology Metal. 9 μm and 10 μm Cu foils (99.99%) were bought from JingLiang Copper and YiGe Metal Material, respectively. Ti foil (99.999%) was bought from Haiyuan Research Metal. Stainless steel foil was bought from Lizhong Metal. Carboxymethyl cellulose sodium (CMC) powder was bought from the Nanjing Modges Energy Technology Co., LTD. Polyvinylidene fluoride (PVDF) powder was bought from Taiyuan Power Source Technology Co., LTD. Acetylene black was bought from Cyberchem Materials. Zinc fluoride (ZnF_2_, 99%) was bought from Energy Chemical. Ultrapure water was obtained from the Milli-Q water purification system.

***Preparation of the Cu@CMC-ZnF_2_ foil, the Cu@PVDF-ZnF_2_ foil and the Cu@CMC foil:*** The glass bottle was initially filled with 6 mL of ultrapure water, followed by the addition of 80 mg of CMC powder. To ensure thorough mixing, the mixture was magnetically stirred at 200 rpm. Subsequently, 720 mg of ZnF_2_ powder was introduced and continued to be stirred at room temperature for approximately 12 hours at 200 rpm to form a homogeneous CMC-ZnF_2_ slurry. Ultrasound treatment was applied to eliminate any minute bubbles in the slurry for a duration of 15 minutes. Magnetic stirring was performed briefly at room temperature after ultrasound treatment. Prior to use, the 9 μm copper foil substrate was washed with ethyl alcohol and dried. Approximately 2-3 mL of slurry were dispensed onto the copper foil using a pipette before coating. A scraper measuring 200 μm in thickness was then used to evenly distribute the CMC-ZnF_2_ slurry onto the surface of the copper foil (9 μm). The coated foil underwent air drying for approximately 1-3 hours before being transferred onto an adjustable heating table set at 80℃ for about an hour until Cu@CMC-ZnF_2_ foil formation is complete.​ The preparation method for Cu@PVDF-ZnF_2_ foils closely resembles that described above; however, NMP replaces ultrapure water and PVDF substitutes CMC as materials used in this process stepwise alteration occurs accordingly. The preparation method for the Cu@CMC foil follows a similar procedure as the Cu@CMC-ZnF_2_ foil but excludes the addition and stirring of ZnF_2_.

***The electroplating of Cu@Zn foils and the preparation of Cu@Zn@CMC-ZnF_2_ foils.*** Cu@Zn foils were prepared using an automatic electroplating device (Figure 1). Since the selected motor speed should not be too slow, an alternating scheme of “electroplating - moving” was adopted here to achieve continuous electroplating (Figure S2). The area of each electroplating was limited by a baffle to a square with a side length of 10 cm, resulting in a total plating area of 100 cm^2^. The copper foil used for electroplating had a thickness of 10 μm and width of 10 cm. The electroplating current was 6 A and the current density was 60 mA cm^-2^. The zinc foil served as the positive electrode while the copper foil acted as the negative electrode, facilitating the flow of zinc ions from the zinc sheet side to the copper foil side through application of positive voltage and current. The vertical distance between these two electrodes was approximately 19 mm. The electrolyte used for electroplating was 2 _M_ ZnSO_4_ aqueous solution. The duration of each plating was about 8.05 minutes, that is, the capacity of deposited zinc was about 8.05 mAh cm^-2^. Prior to use, the Cu@Zn foil obtained by electroplating was washed using ultra-pure water and dried. Cu@Zn@CMC-ZnF_2_ foils were prepared by coating CMC-ZnF_2_ slurry onto the surface of Cu@Zn foils following similar procedures as described above for Cu@CMC-ZnF_2_; however, omitting final heating at 80℃ to minimize ZnO formation.

***Preparation of aqueous electrolyte:*** The particles of ZnSO_4_·7H_2_O weighing 5.74 g and ZnF_2_ powder weighing 82.7 mg were added to 7.48 mL of ultrapure water and stirred thoroughly to form an electrolyte solution containing 10 mL of water. The resulting electrolyte comprised a solution with a concentration of 2 _M_ ZnSO_4_, supplemented with 0.08 _M_ ZnF_2_. The particles of ZnSO_4_·7H_2_O weighing 5.74 g was added to 7.48 mL of ultrapure water and stirred thoroughly to form an 2 _M_ ZnSO_4_ electrolyte solution containing 10 mL of water.

***Preparation of Zn_0.25_V_2_O_5_·nH_2_O (ZnVO) nanoribbons and ZnVO cathodes:*** The ZnVO nanoribbons were synthesized based on previous studies. A mixture of 2 mmol V_2_O_5_ in a solution of 50 mL water/acetone (volume ratio 15:1) was prepared, followed by the addition of 1.3 mmol zinc acetate dihydrate and thorough stirring. The resulting mixture was then transferred to a sealed Teflon container and subjected to a temperature of 200℃ for a duration of 72 hours for synthesis. The obtained product underwent repeated washing with ultrapure water and acetone before being dried at 60℃ for 24 hours. The product was ground in an agate mortar for no less than 20 minutes before use. The cathode was obtained by coating the cathode slurry onto the surface of a thin carbon fiber felt and then drying it overnight in a vacuum oven at 80℃. The preparation process for the cathode slurry was as follows: NMP (4 mL) was added to a glass bottle, followed by adding PVDF (80 mg), and stirring for 6 hours. Then, acetylene black (160 mg) was added and stirred for 1 hour. Finally, ZnVO (560 mg) was added and stirred for 12 hours. ​The mass of the thin carbon fiber felt was approximately 3 mg cm^-2^. After subtracting the mass of the carbon fiber felt, the mass ratio of active material in the cathode was 70%. The load mass of ZnVO was approximately 5 mg cm^-2^ when using a 400 μm scraper.

***Preparation of self-supporting ZnVO cathodes:*** The obtained Zn_0.25_V_2_O_5_·nH_2_O nanoribbons were first uniformly mixed with acetylene black at a mass ratio of 15:3 for 5 minutes. The PTFE emulsion was added to the blended powder, in which the PTFE accounted for one-fifth of the mass of Zn_0.25_V_2_O_5_·nH_2_O. An appropriate amount of isopropanol was added to the mixture. Stir continuously until a doughy mixture forms. The resulting mixture was then rolled multiple times using a roll press to fabricate the cathode sheet. After air drying at room temperature, the cathode sheet was transferred to a 60 ℃ oven for complete removal of isopropanol. The cathode sheet was punched into 11 mm diameter discs for use. The load mass of Zn_0.25_V_2_O_5_·nH_2_O accounts for five-sevenths of the total mass of cathode and is approximately 20 mg cm^-2^.

***Assembly of coin cells:*** The coin cells used CR2025 as battery cases. Battery cases, gaskets and springs were ultrasonically cleaned with ethanol and completely dried before battery assembling. The Zn foils were sanded with sandpaper to remove the surface oil and oxide layer, and then cleaned with alcohol. From bottom to top, the assembly sequence of Cu||Zn half cells was the positive case, the Cu foil (9 μm) or the Cu foil with coating layer (*Φ*19 mm), the *Φ*16 mm GF separator, the *Φ*12 Zn foil with a thickness of 200 μm, 500 μm gasket (diameter < 16 mm), the 1.1 mm spring and the negative case. The assembly sequence of Zn||Zn symmetrical cells was the positive case, the 10 μm Zn foil or the Cu@Zn foil or the Cu@Zn@CMC-ZnF_2_ foil with *Φ*16 mm, the *Φ*19 mm GF separator, the 10 μm Zn foil or the Cu@Zn foil or the Cu@Zn@CMC-ZnF_2_ foil with *Φ*12 mm, the 1000 μm gasket (diameter < 16 mm), the 1.1 mm spring and the negative case. The assembly sequence of the Zn|GF|ZnVO full cell was the positive case, the *Φ*11 mm cathode foil, the *Φ*19 mm GF separator, the *Φ*16 mm anode foil, the 1000 μm gasket, the 1.1 mm spring and the negative case. The assembly sequence of the Zn|PP|ZnVO full cell was the positive case, the *Φ*11 mm self-supporting cathode foil, the *Φ*19 mm PP separator, the *Φ*16 mm anode foil, the 500 μm gasket, the 1.1 mm spring and the negative case. The volume of electrolyte was ≈ 100 μL per cell. The seal pressure was more than 50 kg cm^-2^. These batteries rest at room temperature for ≈ 2 hours before testing. The full batteries are charged to 1.7 V before cycling.

***Assembly of pouch cells:*** Two packing materials were used for the pouch cells, namely a transparent vacuum bag and an opaque aluminum plastic film. The packaging of the pouch battery uses the vacuum pre-sealing machine of Hefei Kejing Material Technology Co., LTD. The packaging temperature of the transparent vacuum bag is 135℃, while the aluminum-plastic film is 170℃. The pole ears of the 2×2 cm Zn||Zn pouch cell in Figure 5d were stainless steel foils with a width of 1 cm and a thickness of 10 μm. The amount of electrolyte added to the pouch cell was 50 μL cm^-2^ calculated based on the separator area. Considering the high horizontal resistance of the thin carbon fiber felt utilized as the ZnVO cathode substrate, a Ti foil with minimal horizontal resistance was employed as the positive current collector on the cathode side in the full cell. The assembly sequence of full cells was the Ti foil, the ZnVO cathode foil, the GF separator and the anode foil. Among them, the Ti foil and the anode directly acted as the pole ears.

***Electrochemical measurements:*** All batteries were tested on the Lanhe battery test system, and the room temperature was controlled at ≈ 25ºC through the air conditioning. The EIS and Tafel curves were tested using the Bio-Logic VSP Five-channel Electrochemical Workstations. The frequency range of the EIS test is 100 kHz to 0.1 Hz for symmetrical cells and 200 kHz to 0.01 Hz for full cells. In addition, the cells for comparison are installed at the same time to reduce interference.

***Characterization:*** The SEM and EDS images were captured using the ZEISS Auriga SEM/FIB Crossbeam System. The accelerating of 5 kV and 20 kV were used for SEM images, and 20 kV was used for EDS measurement. Contact angles were tested by Chengde Dingsheng JY-82C video contact Angle tester. Rigaku Miniflex 600 was used to measure the XRD patterns. The scanning mode is 1D scan, the scanning step is 0.02º, and the scanning speed is 20º min^-1^.

1. Experimental Figures and Tables

**
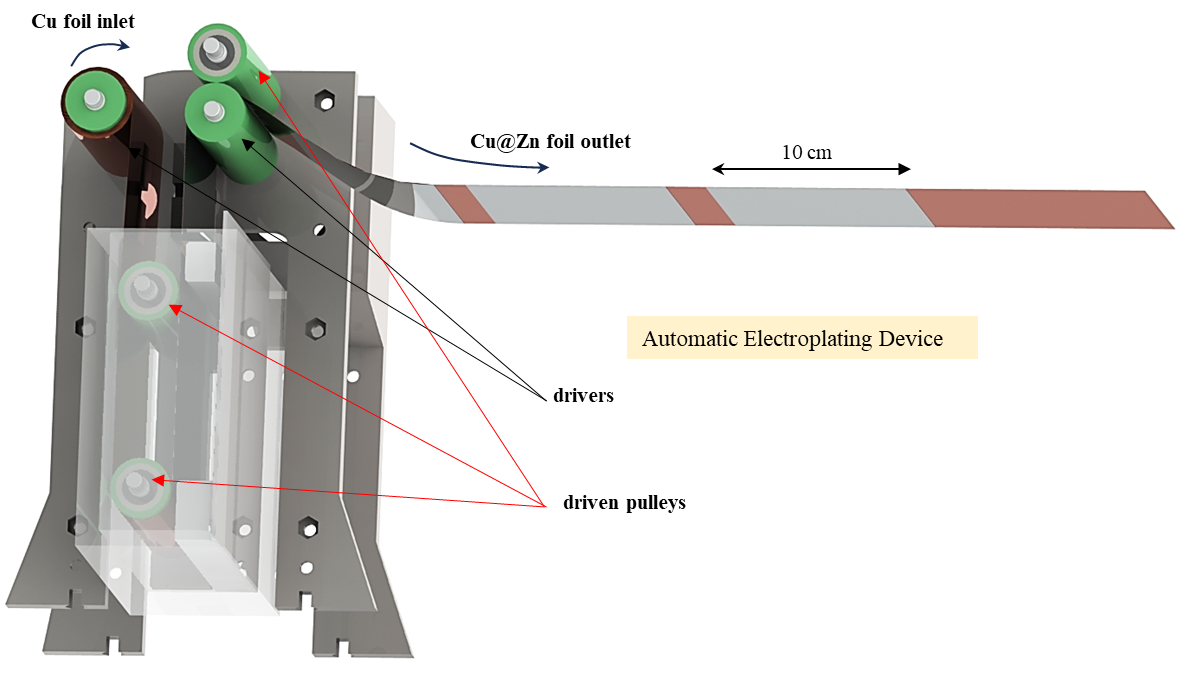
**

**Figure S1** ​A 3D structure diagram of an automatic electroplating device.


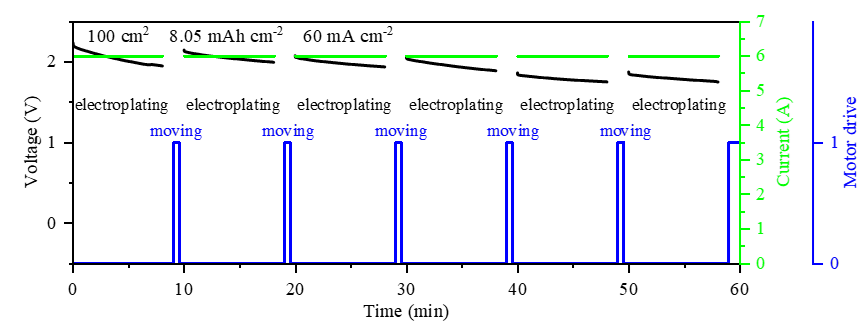


**Figure S2** ​Electroplating voltage, electroplating current and motor drive during the operation of an automatic electroplating device.


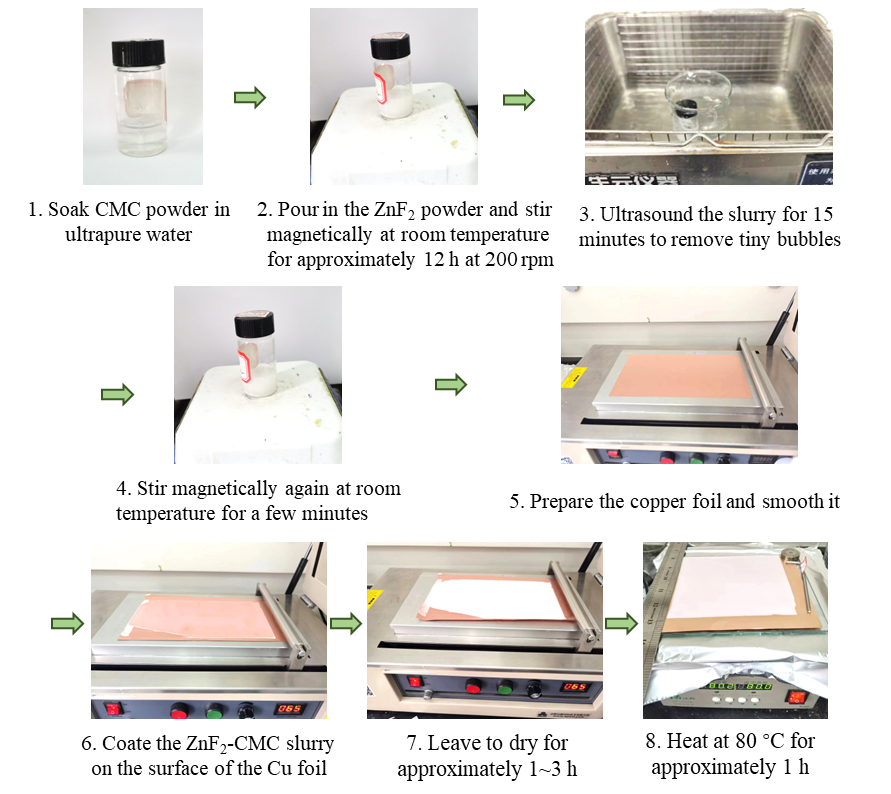


**Figure S3** Photographs depicting the preparation process of the Cu@CMC-ZnF_2_ foil.


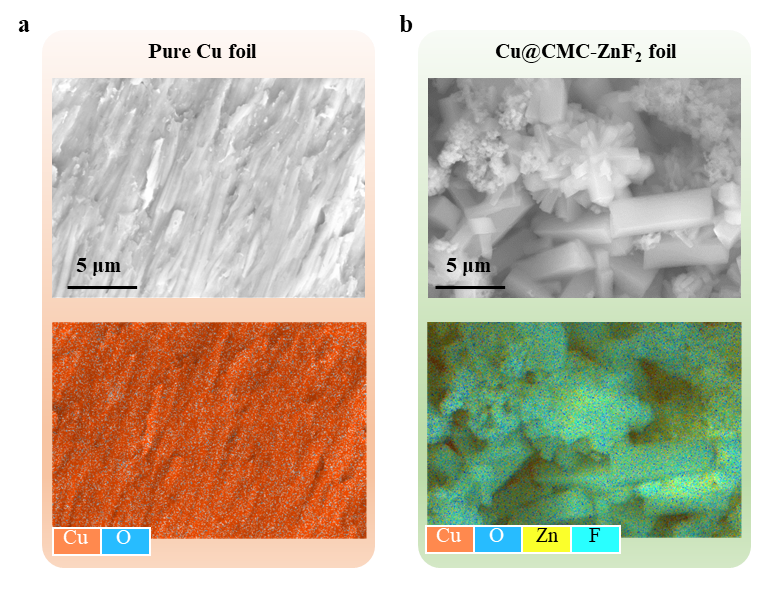


**Figure S4** SEM images and corresponding EDS mappings of (a) the pure Cu foil and (b) the Cu@CMC-ZnF_2_ foil.


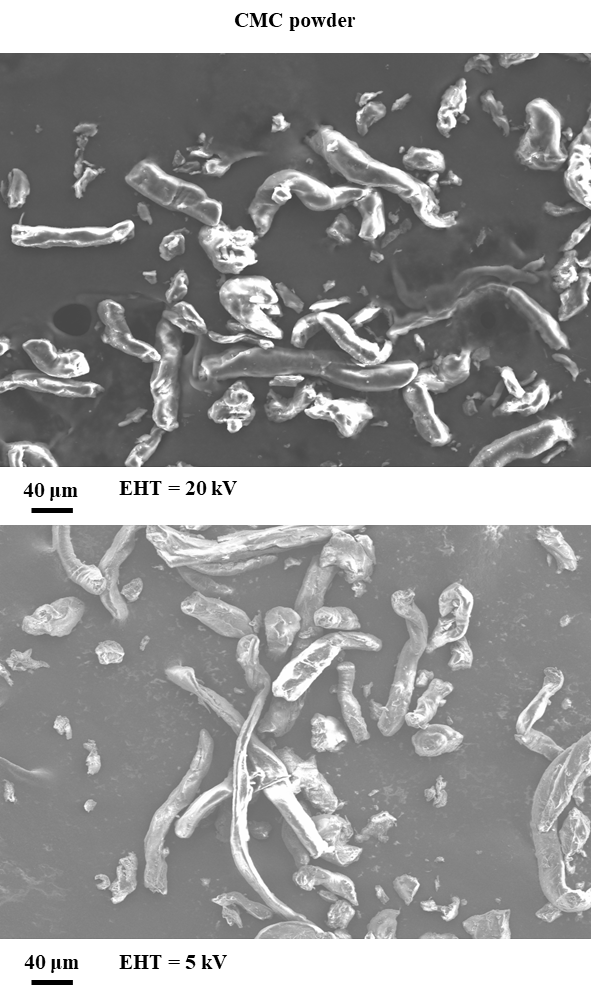


**Figure S5** SEM images of CMC powder.


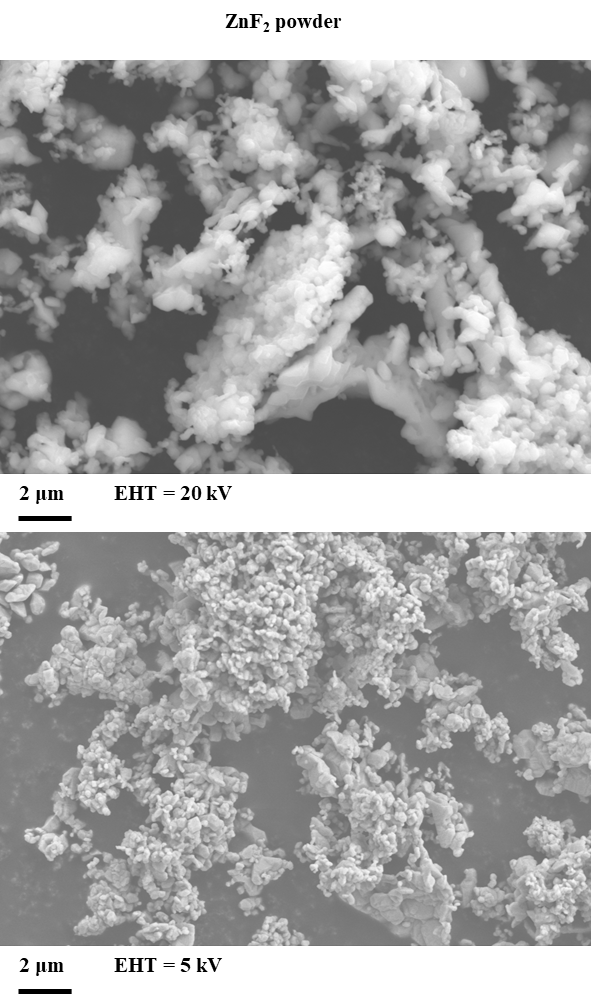


**Figure S6** SEM images of ZnF_2_ powder.


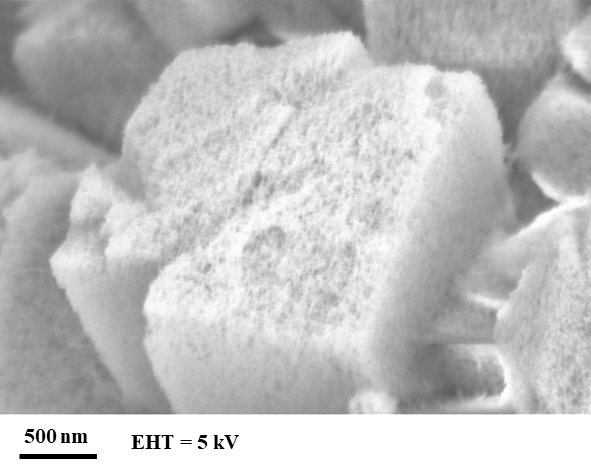


**Figure S7** A SEM image of the terminal face of a CMC prism in the CMC-ZnF_2_ coating.


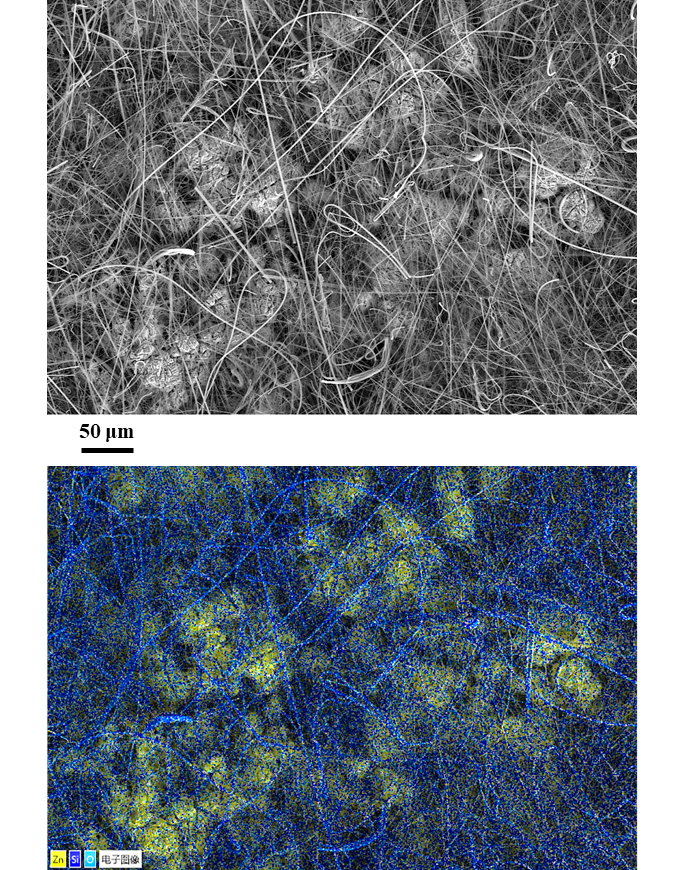


**Figure S8** SEM image and corresponding EDS image of the non-uniform dendritic zinc deposition on a Cu foil at 5 mA h cm^-2^.


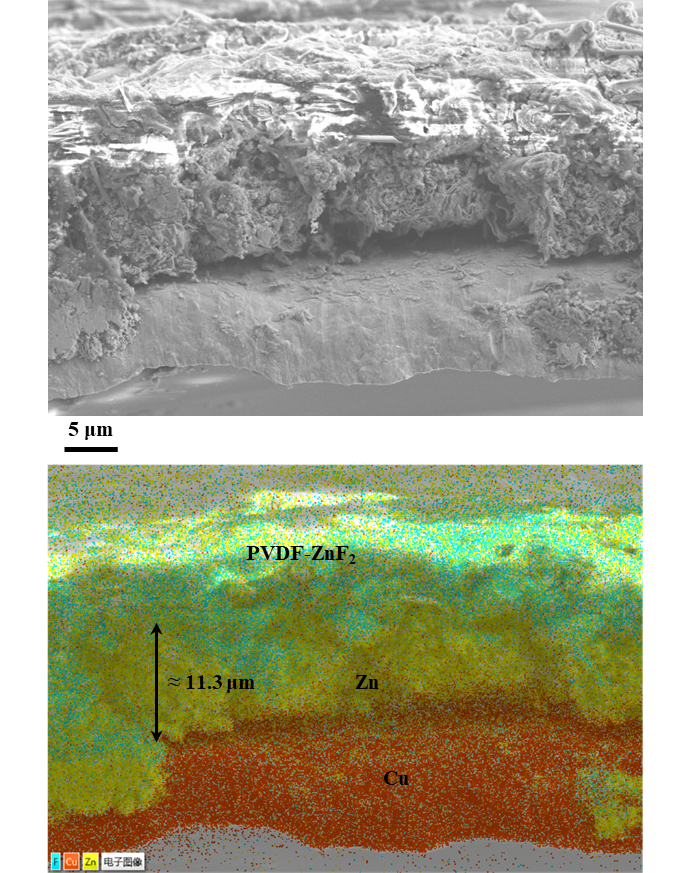


**Figure S9** SEM image and corresponding EDS image of non-uniform zinc deposition at 5 mA h cm^-2^ in a Cu@PVDF-ZnF_2_ foil.


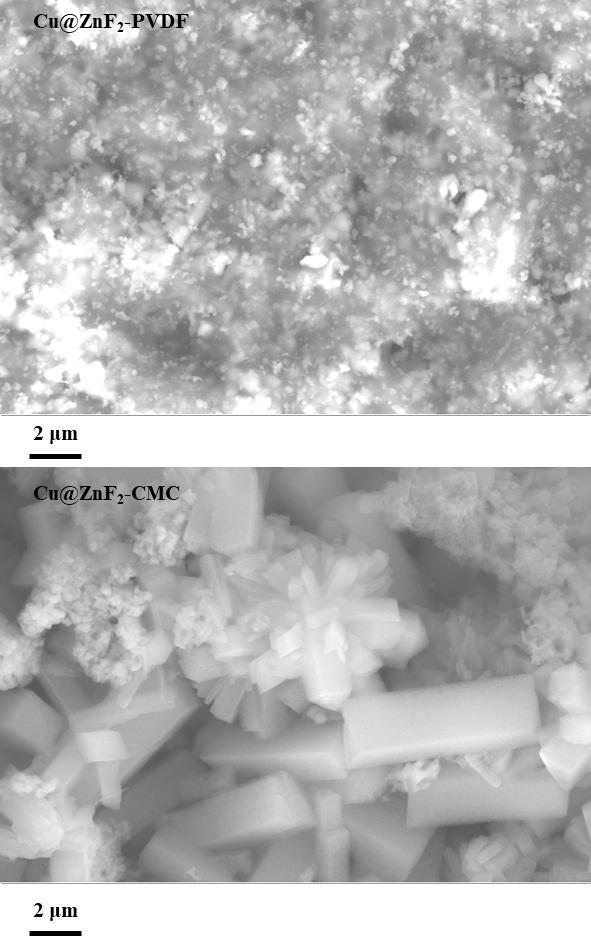


**Figure S10** Comparison of SEM images of Cu@ZnF_2_-PVDF and Cu@CMC-ZnF2 foils.


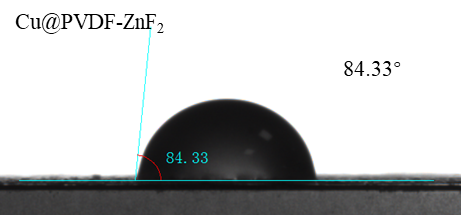


**Figure S11** Contact angle of Cu@PVDF-ZnF_2_.


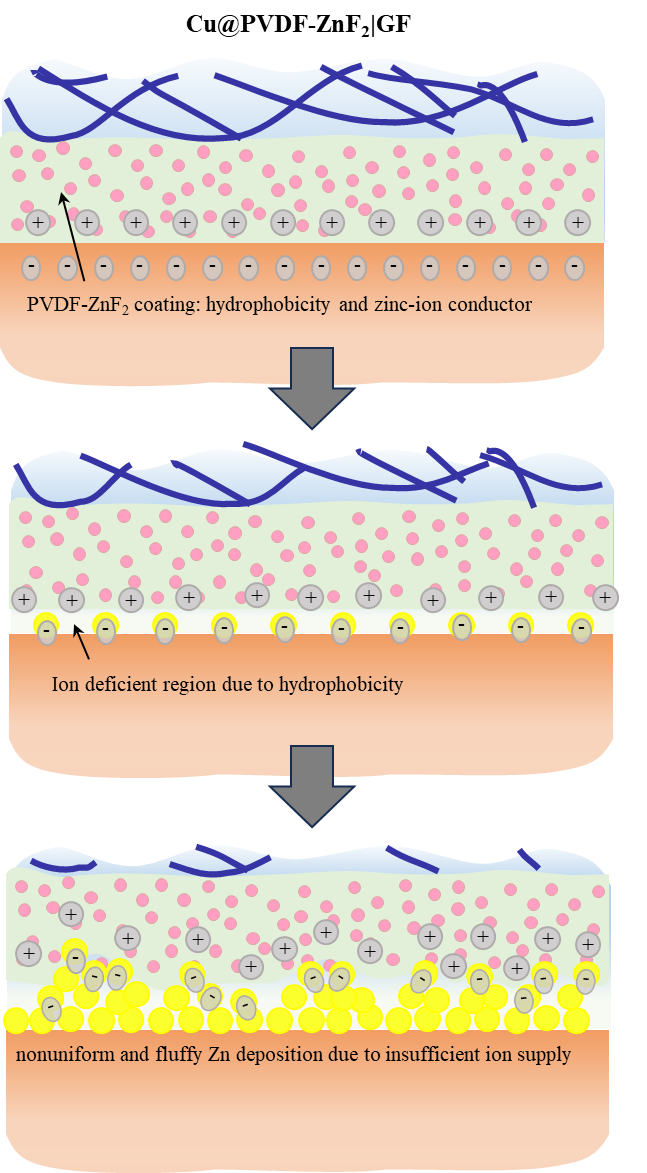


**Figure S12** Schematic illustrations of zinc deposition evolution under the change of electric field and ionic field at Cu|PVDF-ZnF_2_@GFs interface.


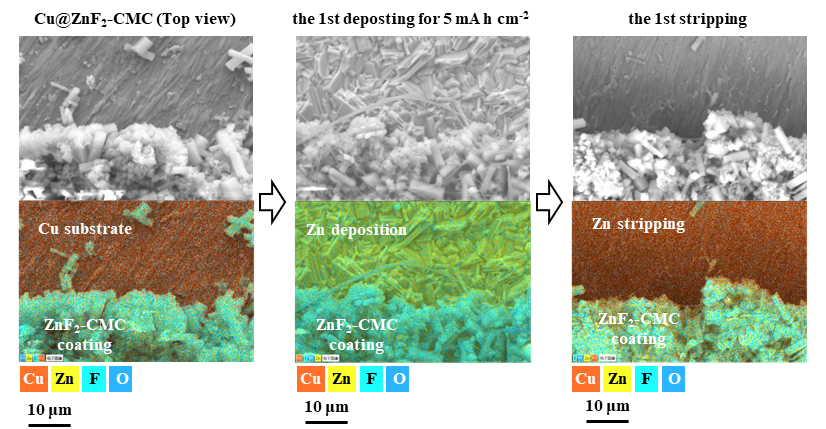


**Figure S13** SEM images and corresponding EDS images depicting surface morphological changes of Cu@CMC-ZnF_2_ in the first cycle at 10 mA cm^-2^ and 5 mA h cm^-2^.


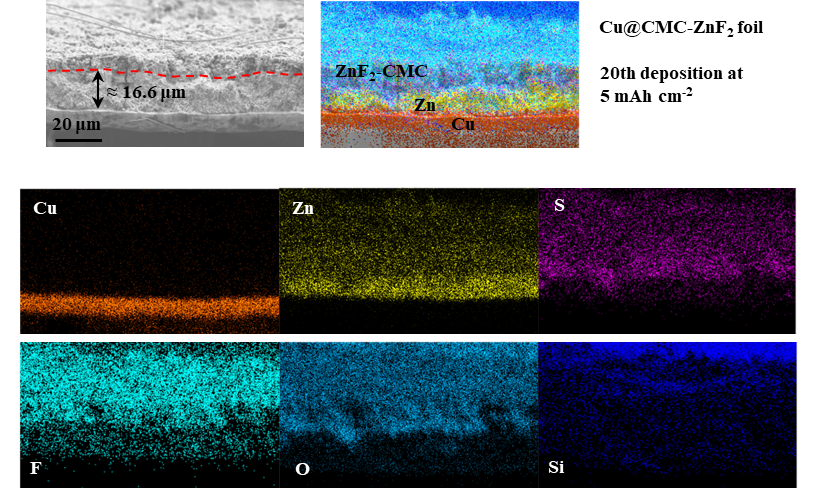


**Figure S14** SEM images and corresponding EDS images of Cu@CMC-ZnF_2_ after the 20th deposition at 10 mA cm^-2^ and 5 mA h cm^-2^.


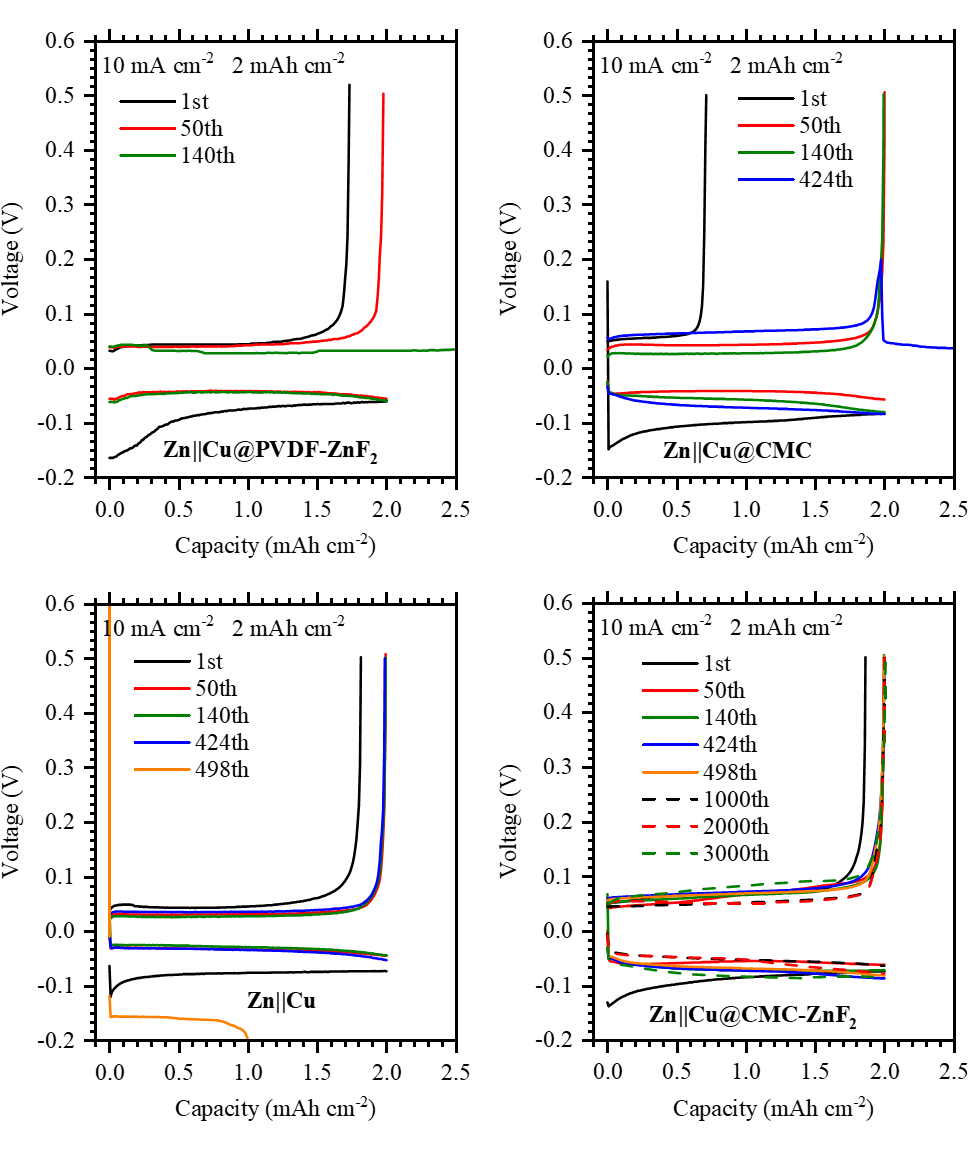


**Figure S15** The charge and discharge curves of Zn||Cu half-cells at 10 mA cm^-2^ and 2 mAh cm^-2^ during different cycles.


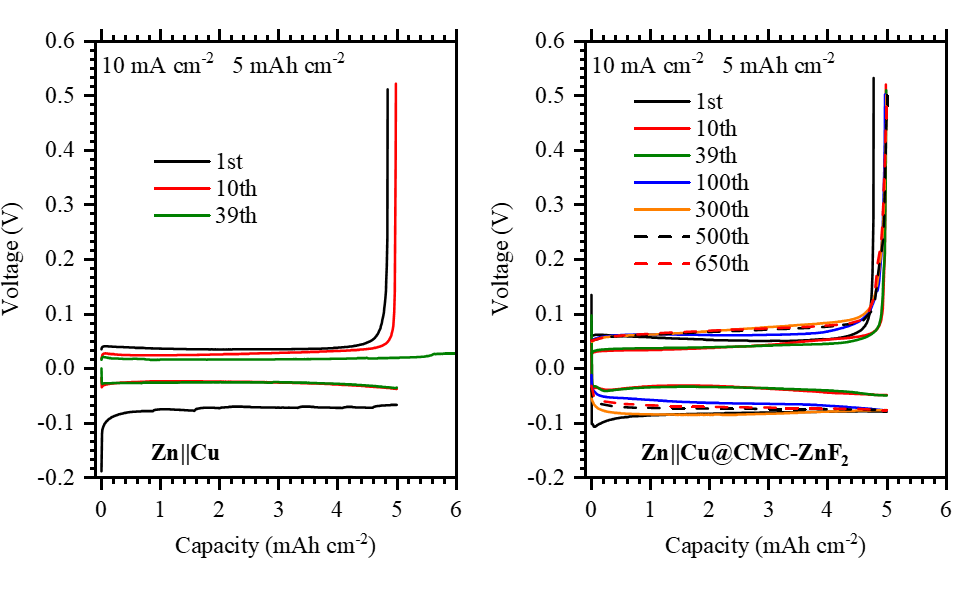


**Figure S16** The charge and discharge curves of Zn||Cu half-cells at 10 mA cm^-2^ and 5 mAh cm^-2^ during different cycles.

**Figure S17** XRD pattern of Cu@Zn@ZnF_2_-CMC foils under various cycle numbers at 5 mAh cm^-2^.


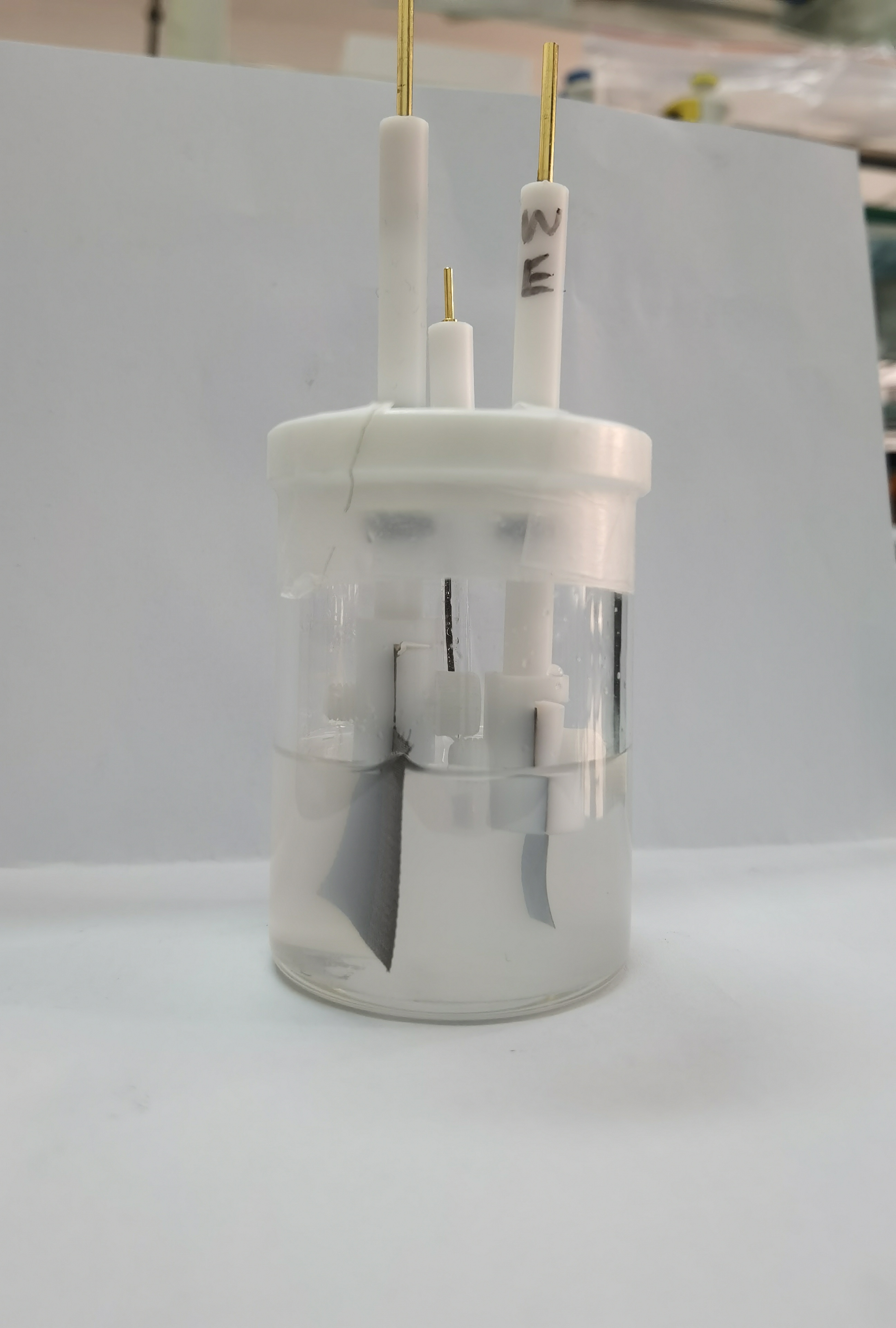


**Figure S1****8** Photo of a three-electrode test system.


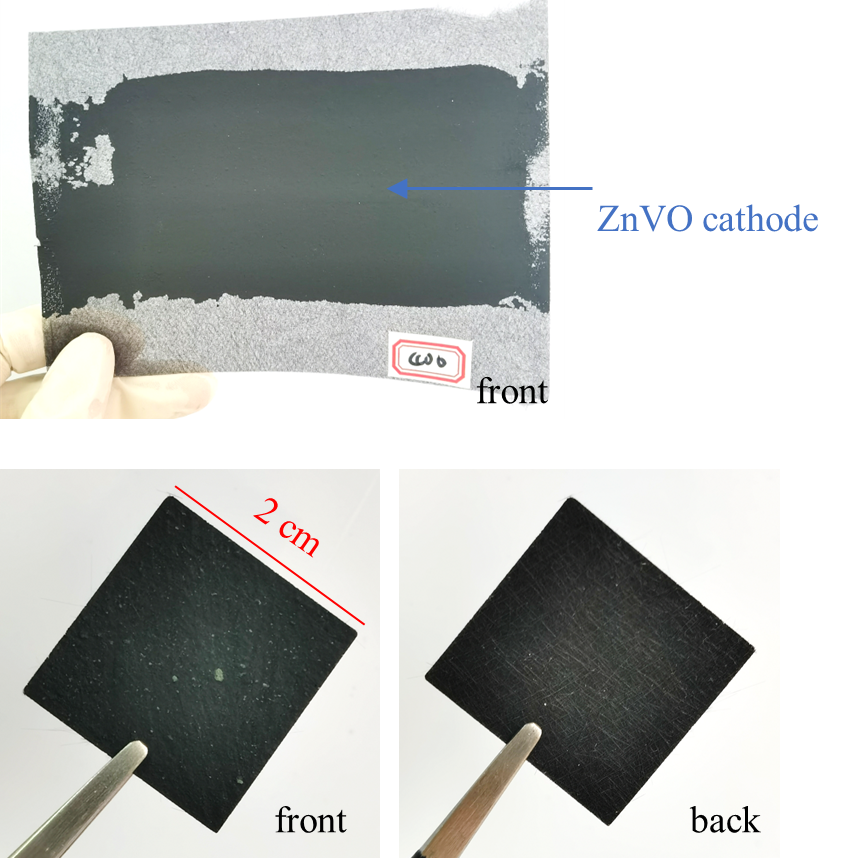


**Figure S19** Photos of ZnVO cathodes.


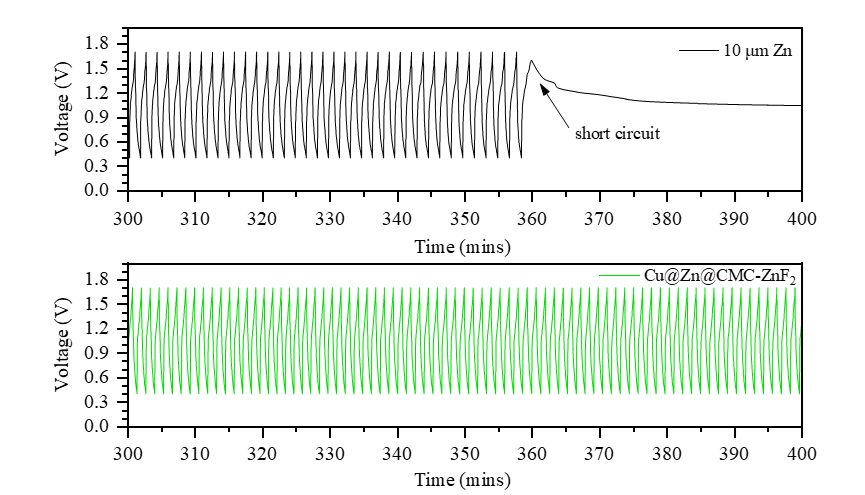


**Figure S20** Comparison of voltage curves between Zn||ZnVO cells using a 10 μm Zn anode and a Cu@Zn@CMC-ZnF_2_ anode at 300-400 minutes.


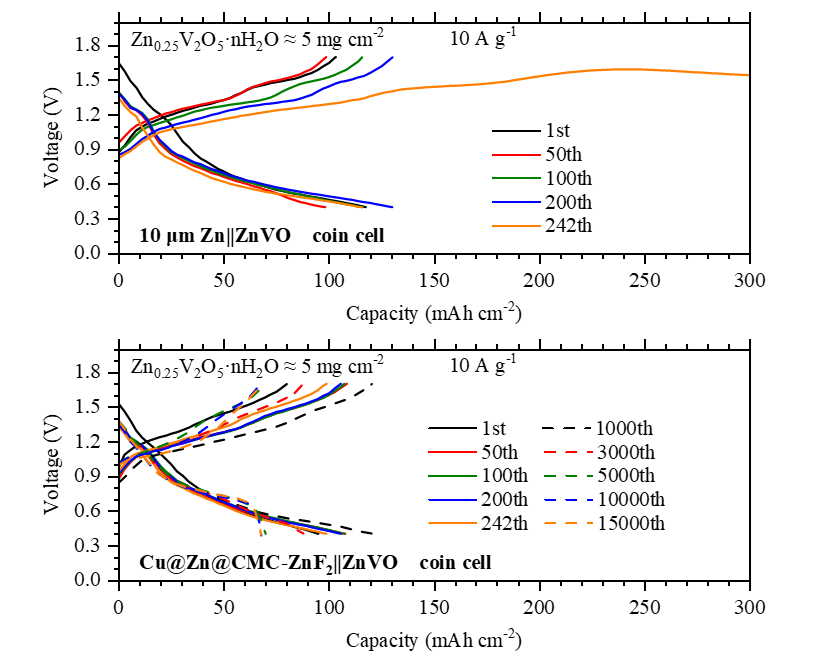


**Figure S21** The charge and discharge curves of Zn||ZnVO coin cells at different cycles.


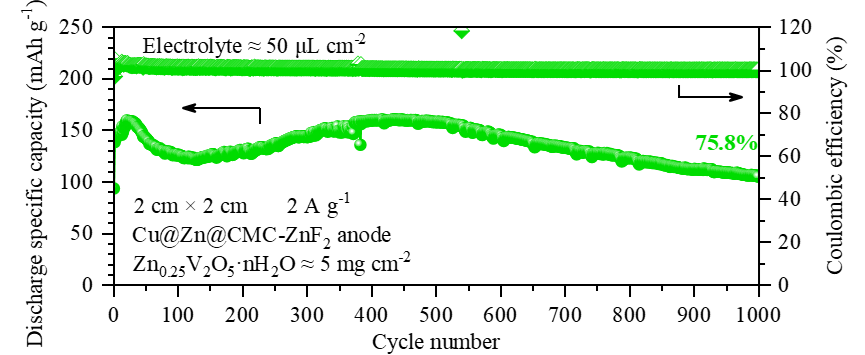


**Figure S22** Discharge specific capacity and Coulombic efficiency of the Cu@Zn@CMC-ZnF_2_||ZnVO pouch cell of 2×2 cm.


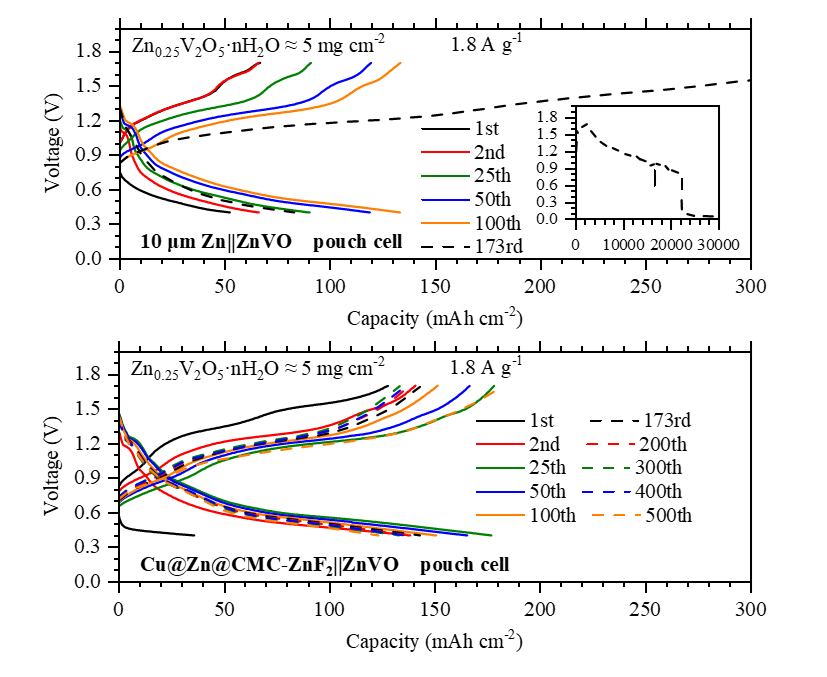


**Figure S23** The charge and discharge curves of Zn||ZnVO pouch cells of 5×5 cm at different cycles.


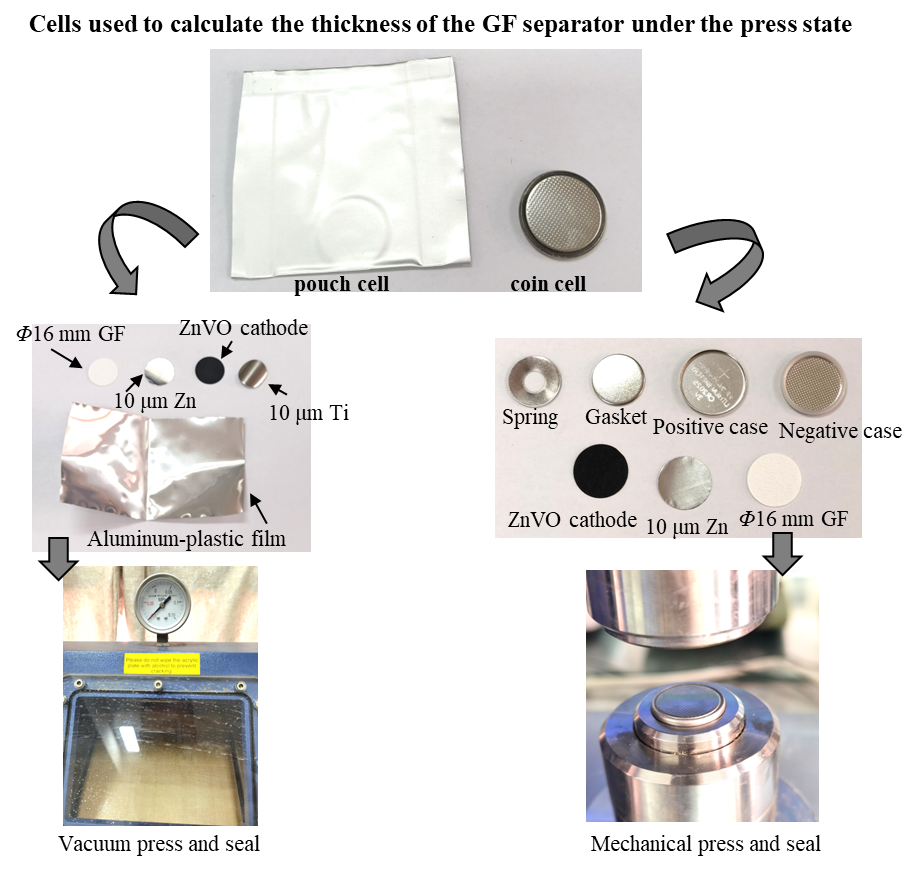


**Figure S24** The photos of cells used to calculate the thickness of the GF separator under the press state.


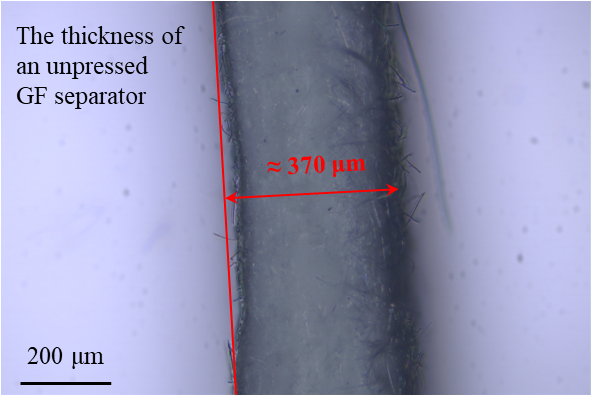


**Figure S25** A photo taken with an optical microscope used to measure the thickness of an unpressed GF separator.


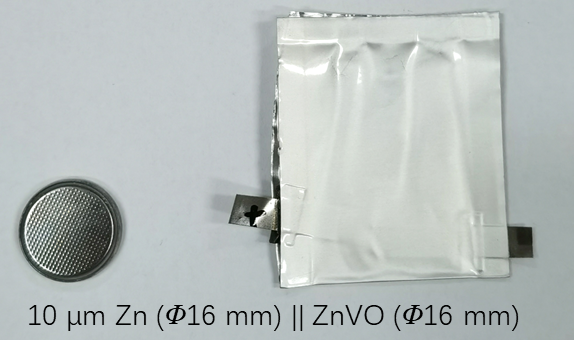


**Figure S26** ​A Photo of full cells used to test the EIS curves.

**Table S1** The mass and theoretical capacity of zinc foils with various thicknesses.

| Materials | Mass (mg cm^-2^) | Theoretical capacity (mAh cm^-2^) | ​DOD^#^  (paired with a cathode possessing a capacity of 5 mAh cm^-2^) |
| --- | --- | --- | --- |
| 10 μm Zn foil | ≈ 9.82* | 9.82 mg cm^-2^ × 820 mAh g^-1^ × 10^-3^ ≈ 8.05 | 5 / 8.05 × 100% ≈ 62.1% |
| 20 μm Zn foil^[1]^ | ≈ 15.93^[1]^ | 15.93 mg cm^-2^ × 820 mAh g^-1^ × 10^-3^ ≈ 13.06 | 5 / 13.06 × 100% ≈ 38.3% |
| 30 μm Zn foil | ≈ 19.64* | 19.64 mg cm^-2^ × 820 mAh g^-1^ × 10^-3^ ≈ 16.10 | 5 / 16.10 × 100% ≈ 31.1% |
| 200 μm Zn foil | ≈ 107.15* | 107.15 mg cm^-2^ × 820 mAh g^-1^ × 10^-3^ ≈ 87.86 | 5 / 87.86 × 100% ≈ 5.7% |

* ​This data was obtained by dividing the mass obtained by weighing three 16 mm diameter zinc foils at room temperature by the area of the zinc foil.

^#^ DOD can be obtained by dividing the usage capacity or discharge capacity by the theoretical capacity.

**Table S2** Referenced 2*θ* values of Cu crystal planes in PDF#04-0836.

| Cu (*hkl*) | 2*θ* |
| --- | --- |
| Cu (111) | 43.297 |
| Cu (200) | 50.433 |
| Cu (220) | 74.13 |
| Cu (311) | 89.93 |

**Table S3** Referenced 2*θ* values of Zn crystal planes in PDF#04-0831 and their angles between normal vectors of crystal planes and the substrate.

| Zn (*hkl*) | 2*θ* | ​Angle between the normal vector of the crystal plane and the substrate |
| --- | --- | --- |
| Zn (002) | 36.296 | 90 |
| Zn (100) | 38.992 | 0 |
| Zn (101) | 43.231 | 45 |
| Zn (102) | 54.336 | 63.43 |
| Zn (103) | 70.056 | 71.57 |
| Zn (110) | 70.66 | 0 |
| Zn (004) | 77.027 | 90 |
| Zn (112) | 82.102 | 54.74 |
| Zn (200) | 83.765 | 0 |
| Zn (201) | 86.557 | 26.57 |

**Table S4** The mass per unit area of some materials.

| Materials | Mass (mg cm^-2^) |
| --- | --- |
| 10 μm Zn foil  (≈ 8.05 mAh cm^-2^ Zn) | ≈ 9.82 |
| 9 μm Cu foil | ≈ 9.38 |
| 10 μm Cu foil | ≈ 11.05 |
| Cu@Zn@CMC-ZnF_2_ foil (10 μm Cu and ≈ 8.05 mAh cm^-2^ Zn) | ≈ 22.18 |

**Table S5** ​The thickness of the components and the GF separator in the coin cell.

|  | Coin cell  (without electrolyte) | Coin cell  (with 100 μL electrolyte) |
| --- | --- | --- |
| Total thickness | 2.63 mm | 2.59 mm |
| Central thickness of positive case | 0.25 mm | 0.25 mm |
| Central thickness of negative case | 0.25 mm | 0.25 mm |
| Zn foil | 0.01 mm | 0.01 mm |
| ZnVO cathode | 0.16 mm  (Before pressing: 0.23 mm) | 0.16 mm  (Before pressing: 0.23 mm) |
| Gasket | 1.02 mm | 1.03 mm |
| Spring | 0.82 mm (Before pressing: 1.1 mm) | 0.76 mm (Before pressing: 1.1 mm) |
| GF | 0.12 mm / 120 μm (2.63 mm – 0.25 mm – 0.25 mm – 0.01 mm – 0.16 mm – 1.02 mm – 0.82 mm = 0.12 mm) | 0.13 mm / 130 μm (2.59 mm – 0.25 mm – 0.25 mm – 0.01 mm – 0.16 mm – 1.03 mm – 0.76 mm = 0.13 mm) |

**Table S6** ​The thickness of the components and the GF separator in the pouch cell.

|  | Pouch cell  (without electrolyte) | Pouch cell  (with 100 μL electrolyte) |
| --- | --- | --- |
| Total thickness | 0.69 mm | 0.66 mm |
| Two layers of aluminum-plastic film | 0.21 mm | 0.21 mm |
| Zn foil | 0.01 mm | 0.01 mm |
| Ti foil | 0.01 mm | 0.01 mm |
| ZnVO cathode | 0.20 mm  (Before pressing: 0.23 mm) | 0.19 mm  (Before pressing: 0.23 mm) |
| GF | 0.26 mm / 260 μm (0.69 mm – 0.21 mm – 0.01 mm – 0.01 mm – 0.20 mm = 0.26 mm) | 0.24 mm / 240 μm (0.66 mm – 0.21 mm – 0.01 mm – 0.01 mm – 0.19 mm = 0.24 mm) |

Reference:

[1] W. Du, S. Huang, Y. Zhang, M. Ye, C. C. Li, *Energy Storage Mater.* **2022**, *45*, 465.
